# TGFβ inhibition and mesenchymal to epithelial transition initiation by *Xenopus* egg extract: first steps towards early reprogramming in fish somatic cell

**DOI:** 10.1038/s41598-023-36354-3

**Published:** 2023-06-20

**Authors:** Nathalie Chênais, Aurelie Le Cam, Brigitte Guillet, Jean-Jacques Lareyre, Catherine Labbé

**Affiliations:** 1 INRAE, UR1037 LPGP, Fish Physiology and Genomics, Campus de Beaulieu, 35000 Rennes, France; 2 Université de Rennes 1, Campus de Beaulieu, 35000 Rennes, France

**Keywords:** Biotechnology, Cell biology, Stem cells

## Abstract

*Xenopus* egg extract is a powerful material to modify cultured cells fate and to induce cellular reprogramming in mammals. In this study, the response of goldfish fin cells to in vitro exposure to *Xenopus* egg extract, and subsequent culture, was studied using a cDNA microarray approach, gene ontology and KEGG pathways analyses, and qPCR validation. We observed that several actors of the TGFβ and Wnt/β-catenin signaling pathways, as well as some mesenchymal markers, were inhibited in treated cells, while several epithelial markers were upregulated. This was associated with morphological changes of the cells in culture, suggesting that egg extract drove cultured fin cells towards a mesenchymal-epithelial transition. This indicates that Xenopus egg extract treatment relieved some barriers of somatic reprogramming in fish cells. However, the lack of re-expression of *pou2* and *nanog* pluripotency markers, the absence of DNA methylation remodeling of their promoter region, and the strong decrease in de novo lipid biosynthesis metabolism, indicate that reprogramming was only partial. The observed changes may render these treated cells more suitable for studies on in vivo reprogramming after somatic cell nuclear transfer.

## Introduction

In fish, somatic cells and particularly fin cells are a convenient source of diploid material for cryopreservation of valuable genetic resources^1^. Such use of somatic cells compensates for the impossibility to cryopreserve fish oocytes and embryos. Besides, fin cells are easy to collect whatever the sex, maturation status or size of the fish, and they are easy to cryopreserve^2,3^. However, regeneration of fish from these highly differentiated cells requires to master nuclear transfer, a technology that is still not reliable enough in fish. Indeed, whereas nuclear transfer with embryonic donor cells yields acceptable development rates^4–8^, only few clones were reported to reach adulthood when the donor cell was taken from adult fish^9–16^. One hypothesis often proposed to explain the low success rate of somatic cell nuclear transfer is the chromatin reprogramming failure (reviewed in mammals^17^). In fish, zebrafish clones at dome stage fail to re-express several genes that are important for chromatin remodeling, translation initiation or cell cycle^18^. More recently, we showed that DNA methylation of several marker genes in goldfish clones failed to match the hypomethylation status of control embryos, and some clones bore the hypermethylated pattern of the donor fin cells^16^. This means that after nuclear transfer, exposure of the somatic chromatin to oocyte factors prior to embryonic genome activation is not sufficient to overcome somatic cell resistance to reprogramming in fish.

Numerous studies in mammals have sought to improve the reprograming ability of donor somatic cells by way of an in vitro pre-reprogramming before nuclear transfer. The ability of metaphase-II (MII) egg factors to ensure chromatin remodeling of sperm and oocyte chromatin following fertilization makes the egg extract an attractive candidate for in vitro reprogramming. Heterologous *Xenopus* eggs at MII stage have been reported to improve the blastocyst rates after nuclear transfer in mouse^19^, ovine^20^ and porcine^21,22^, and although assessed in only few studies, it also increased the development success after implantation or birth^20^. At the molecular level, these heterologous egg extracts have also been shown to induce transcriptional and epigenetic remodeling in mammalian cultured cells. However, such reprogramming of somatic cultured cells is not straightforward and it suffers high variability. Many factors such as the animal species and cell type^23,24^, the culture conditions and egg extract batches or stages^19,24–26^ influenced the extent of somatic cell reprogramming. For example, when considering the expression of pluripotency markers, porcine cells treated with *Xenopus* egg extract only transiently re-expressed *Oct4* over culture time^25,27^ and *Nanog* expression failed to be consistently re-expressed^25,28^ while in mouse, *Oct4* and *Nanog* were both re-expressed^19,29^. Moreover, the extent of reprogramming at the scale of the whole genome is not known. Indeed, all studies are assessing the reprogramming success from candidate genes analysis, and a reprogramming assessment based on all other putative actors of pluripotency is still missing. As a consequence, knowledge on the gene network rewiring upon in vitro reprograming with egg extracts remains elusive.

The question is still open in fish as to whether *Xenopus* egg extract can alter the course of somatic cells in culture, and if this treatment bears the potential to later on improve nuclear transfer in fish species. In a previous study, we demonstrated that goldfish fin cells in primary culture can incorporate *Xenopus* MII-egg extract molecules such as LaminB3 in their nucleus^30^, thus providing evidence that egg factors can reach somatic cells chromatin. It has been described in mammals that the reprogramming effect of *xenopus* egg extracts requires a culture step of the cells, so that they can recover from the treatment and that the new cellular program can induce changes in gene expression^25^. However, the treated cells in our former work were too fragile to be cultured, and the reprogramming consequences of the treatment had been impossible to study. In the present work, we have set up a procedure which allowed the survival and proliferation of the goldfish treated cells in culture. This enabled the analysis of their reprogramming extent. The use of nuclear transfer success as a mean to assess the extent of donor cell reprograming was excluded because of the multiparametric factors at stake in clone development success in fish. Indeed, embryonic failures are a combination of mitotic errors^31^ and gene reprogramming defects^16,18^ whose respective contribution is highly variable between clones. Therefore, the consequences are impossible to discriminate one from the other at the embryonic genome activation stage, when most clones fail to develop^11–13^.

The aim of the present work was to investigate the response of goldfish somatic cells to treatment with *Xenopus* egg extract in culture, and to assess whether this treatment triggered some reprogramming events that would take place ahead of cell collection for nuclear transfer. Changes in gene expression were analyzed by an unbiased microarray approach, and specific networks associated with reprogramming were sought, in relation with the behavioral changes of the cultured cells. At the epigenetic level, changes in DNA methylation pattern of some candidate genes were also explored, namely the *pou2* and *nanog* genes that have differentially methylated promoters between fin cells and embryonic cells^32,33^. Cells from primary fin culture were chosen over cell lines, because the former are closer to the original genetic background that is sought for regeneration of valuable fish genotypes by nuclear transfer.

## Results

### Validation of fin cell exposure to *Xenopus* egg extract

**Figure 1:**
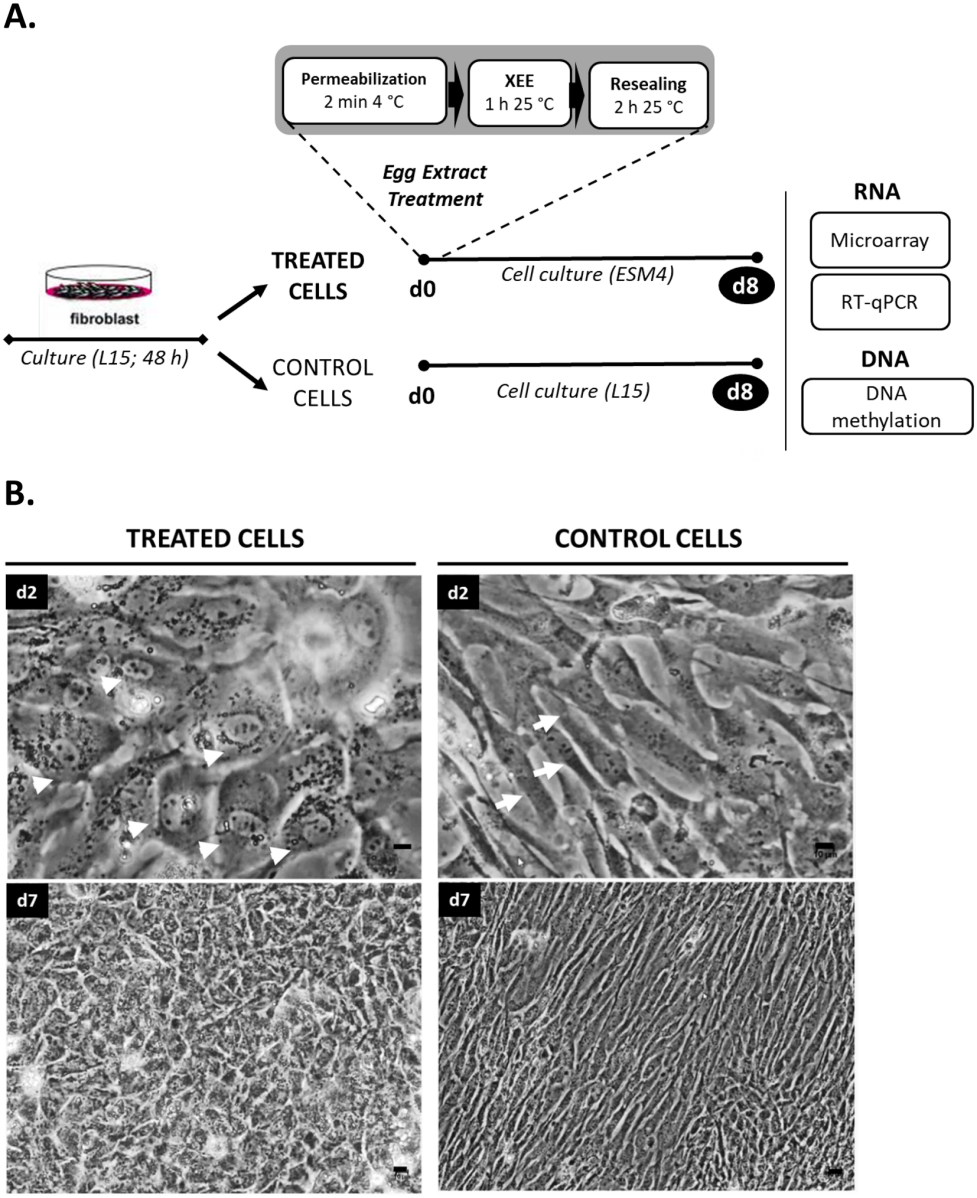
Fish somatic cell treatment with *Xenopus laevis* egg extracts. (**A**) : Summary of the treatment and culture steps. Cell treatment included (i) plasma membrane permeabilization with 30 µg/ mL digitonin, (ii) exposure of the permeabilized cells to egg extracts (XEE) and, (iii) plasma membrane resealing in medium with 2 mM calcium. Treated cells were cultured in ESM4 medium. Control cells originating from the same batches were cultured in L15 medium. (**B) **: Pictures of the treated and control cells over culture time. White arrowheads show treated cells with a cubic shape morphology 2 days after the treatment (d2), contrasting with the elongated (white arrows) control cells. This difference in morphology between treated and control cells was still observed at confluence, 7 days after the treatment (d7). Pictures are representative of three experiments with different cells and egg extract batches. Scale bar = 10 µm.

The mesenchymal cell preparation and treatment that were set up in a previous study^30^ included plasma membrane permeabilization with digitonin, permeabilized cell exposure to egg extract for 1 h, and plasma membrane resealing (Fig. 1A). Penetration of egg factors per se was not assessed here, because this would have required cell fixation. However, all cells displayed the phenotypic characteristics of permeabilized cells and egg extract-treated cells that were described previously^30^: their nuclear membrane was more contrasted after permeabilization than in control cells, their adhesion capacity lessened during egg extract exposure and remained very low during the resealing step and the first 24 h of culture, and they all adopted a round and refracting morphology after resealing. Taken together, our observations indicate that all treated cell batches in the present study did incorporate egg extract.

### The treated cells need a suitable culture medium to survive and proliferate

The culture phase of the treated cells had to be mastered, so that the treated cells could undertake their new cellular program. When the conventional L15 medium was used, we observed that from the second day of culture on, many treated cells were already displaying a cubic shape (Supplementary Fig. S1) that contrasted with the much more elongated control cells (Fig. 1B). However, after 7 days of culture in L15, the treated cell density was decreasing, and many cells were detached from the culture plate (Supplementary Fig. S1), whereas control cells kept proliferating (Fig. 1B). Therefore, we sought for a culture medium that would sustain treated cells survival and proliferation, while maintaining their modified state. To this end, we tested the ESM4 medium enriched with goldfish embryo extracts. After 2 days in this new culture medium, the cubic shape of the treated cells seen in L15 was maintained in ESM4 (Fig. 1B). The elongated shape of the control cells in ESM4 was not changed either (Supplementary Fig. S1), indicating that ESM4 had no effect of its own on the shape of the cultured cells. Most interestingly, the treated cells cultured in ESM4 were able to proliferate over 8 days when they could not in L15 medium (Supplementary Fig. S1). Treated cells in ESM4 showed an increased cell density at day 7, debris and floating cells were no longer observed, and they maintained their specific cubic morphology (Fig. 1B).

### Changes in gene expression eight days after egg extract treatment

#### Clustering of the differentially expressed genes (DEGs)

**Figure 2:**
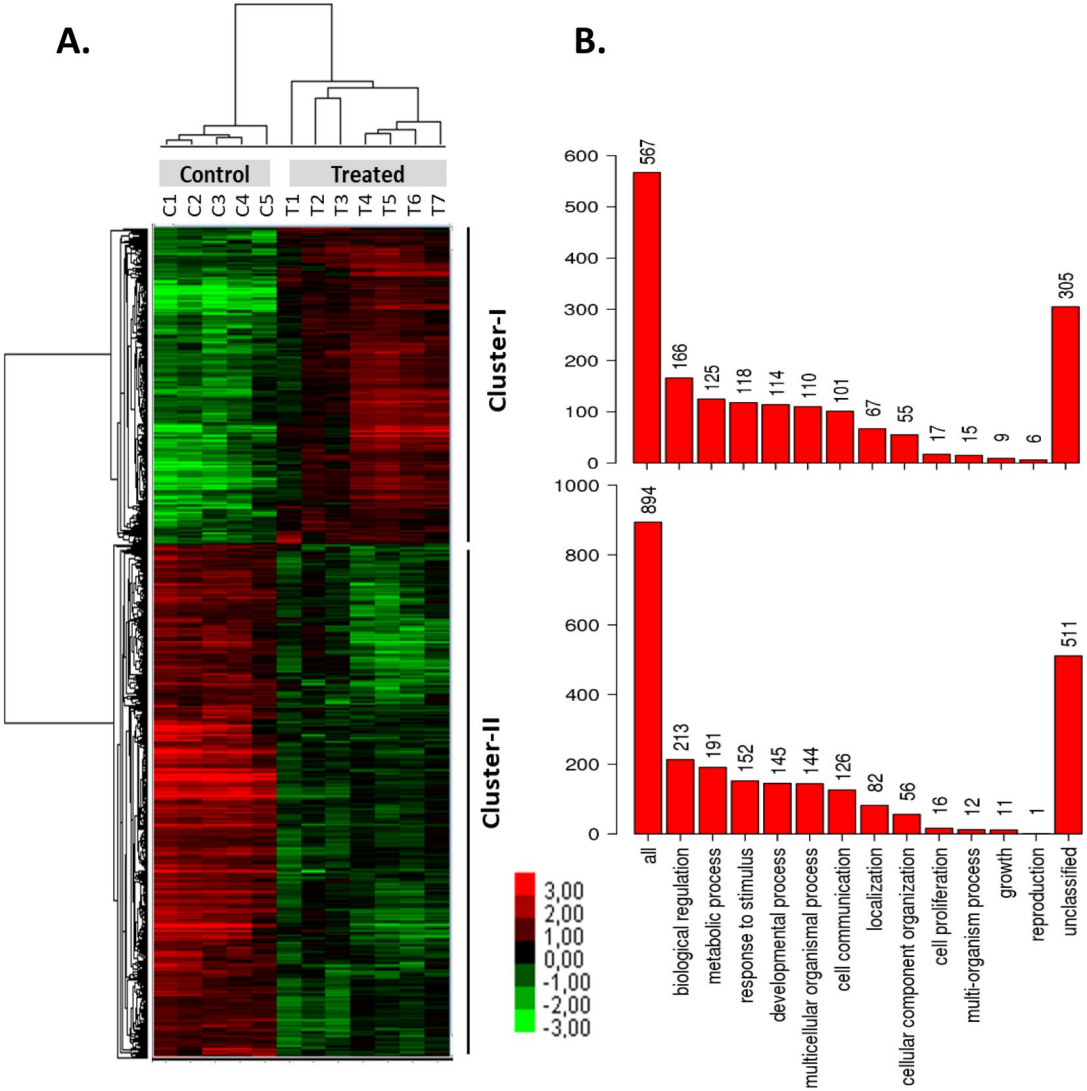
Clustering and gene ontology of the differentially expressed genes after egg extract treatment. (**A**) Heatmap of the hierarchical clustering analysis by unsupervised approach using 52,362 goldfish genes (Java TreeView software; https://bitbucket.org/TreeView3Dev/treeview3/src/master/). Control: control cultured cells (C1-5); Treated: treated cultured cells (T1–7). Each row represents a single gene. Differentially expressed genes (Fold Change > 2; False Discovery Rate (FDR) < 0.05) between treated and control cells are shown on the heatmap (2,286 genes). Two clusters were identified. Cluster-I (872 genes) and cluster-II (1414 genes) contain the genes that were respectively up- and downregulated in treated cells compared to control cells. (**B**) Gene ontology bar chart of the biological process categories after gene ontology (GO) analysis of differentially expressed genes between egg extract-treated and control cells. Distribution of biological process GO in cluster-I and cluster-II (WebGestalt web tool).

Analysis of the microarray data revealed that 2286 goldfish genes out of the 52,362 genes on the microarray were differentially expressed between treated and control cells (fold change > 2). Additionally, hierarchical clustering analysis of differentially expressed genes (DEGs) showed a clear segregation between treated and control samples (Fig. 2A, upper dendrogram). This demonstrates that the treated cells transcriptome was modified by egg extract treatment and that the consequences were detectable after 8 days of culture. Differentially expressed genes between cultured treated and control cells showed a distribution into two clusters on the heatmap (Fig. 2A). Cluster I gathers 872 genes (encompassing 38% of the DEG) that showed upregulation in the treated cultured cells. Cluster II comprises 1 414 genes (62% of the DEG) that were down regulated in the treated cells. Genes in each cluster are listed in Supplementary TableS2.

#### Segregation of the treated samples according to egg-extract batches

Although all treated samples segregated together and showed the same expression profile clustering, it is noteworthy that samples T1 to T3 segregated together, apart from the 4 other samples (T4-T7) (Fig. 2A, upper dendrogram). One possible explanation lies in the egg-extract batches that were used for the different samples. Indeed, the extracts were all prepared from freshly spawned MII stage eggs, each extract being obtained from the spawn of a different female. We cannot exclude that the individual extracts presented some quality variations one from another, notably because of the instability of MII stage in spawned eggs^34^. In order to validate the egg extract stage, we used two MII markers: Greatwall whose phosphorylated forms prevent mitosis/meiosis exit^35^, and Cyclin B whose degradation characterizes mitosis/meiosis exit. Egg extracts arrested at MII stage all displayed a specific western blot profile (supplementary Fig. S2): Greatwall (Gwl) was phosphorylated and stable over incubation time, and cyclin B (CycB) content was high and stable as well. Upon in vitro induction of MII exit by Ca^2+^, Greatwall was successfully dephosphorylated and cyclin B underwent degradation. Contrarily to these well-defined MII egg extracts, some egg extracts showed Greatwall dephosphorylation and Cyclin B degradation, indicating that they had initiated MII exit (MII late stage). Interestingly, the egg extracts used to treat T1 to T3 samples where in MII stage whereas those of T4 to T7 samples had initiated MII exit to some extent (Supplementary Fig. S2). As a conclusion, the sample segregation in the treated cells was likely related to the extract stages (MII and MII-late). This highlights the importance of a careful characterization of the *Xenopus* egg extracts. Although the sample number in each category was low, we still performed a fold change analysis between the two groups. We observed that 83% of the DEGs between MII and MII-late extract groups had low fold changes (< 6), and only 52 genes had fold changes above 6, among which only 9 genes were above 20. No significant or straightforward biological processes were identified via the GO terms analysis, and no marker gene of any specific biological significance emerged from a gene to gene scouting. To conclude, and within the limits of this small sampling, the egg extract stage did not thoroughly affect cellular response, and the two clusters of up- and downregulated genes were observed in all 7 treated cells batches irrespective of the egg extract that was used.

#### Gene ontology (GO) analysis of the differentially expressed genes after egg extract treatment

GO analysis was a perquisite in order to process our DEG list into functions and biological significance. For this purpose, we had first to translate the goldfish gene identifiers into those of the closest species whose genome is well annotated in the GO databases, the zebrafish. This artificially reduced the number of DEG, because the zebrafish did not undergo the genome duplication reported in the *Cyprininae* sub-family, to which goldfish belongs. Only 1533 zebrafish genes (i.e. 67% of total goldfish DEG) were retained for subsequent annotations in GO. Of these, 591 annotated genes were up-regulated (cluster I) and 942 annotated genes were down regulated (cluster II) in the treated cells. GO analysis conducted with the WebGestalt web tool^36^ showed that biological regulation and metabolic process were the most represented terms (Fig. 2B) among biological process GO terms. Surprisingly, no straightforward reprogramming processes such as chromatin remodeling, stem cells, transcription factors, or pluripotency could be emphasized in GO terms after a statistical over-representation analysis. However, several other biological process terms significantly enriched in the GO terms list deserve specific attention.

### Deregulation of TGFβ and Wnt signaling pathways after egg extract treatment

**Figure 3:**
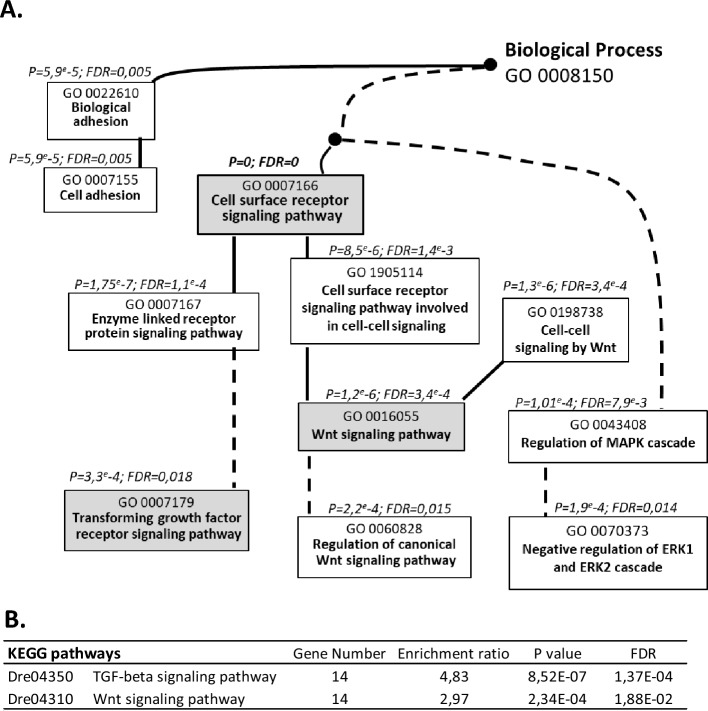
Gene Ontology (GO) flow diagram of the terms related to cell surface receptor signaling pathway (**A**) and KEGG pathways (**B**). The analysis was performed on the cluster of upregulated genes in treated cells (fold change > 2) using WebGestalt web tool. The set of genes spotted on the microarray was used as the reference gene list. A: The black and the dotted lines represent respectively direct and indirect and KEGG pathway (www.kegg.jp/kegg/kegg1.html), p values (P) below 0.05 and false discovery rate (FDR) below 0.05 are indicated. Both A and B highlight the disturbance of the TGFβ and Wnt signaling pathways in response to egg extract treatment.

Our work is reporting gene expression variation, but GO databases and related publications on gene function report mainly protein functions. It is therefore the protein writing nomenclature that will be used in the following sections dedicated to GO interpretation. The most significant GO term obtained from the cluster of up-regulated genes is the cell surface receptor signaling pathway (Fig. 3A). The data mapping showed that this GO term was linked to highly significant child GO terms that are transforming growth factor beta (TGFβ) receptor signaling pathway, and Wnt signaling pathway together with regulation of canonical Wnt signaling pathway. This result was consistent with the KEGG (Kyoto Encyclopedia of Genes and Genomes) analysis performed on the same set of DEG data, that also showed that both TGFβ and Wnt signaling pathways reached a significant level of enrichment among all the database terms (Fig. 3B). To add on to the highlighting of these 2 specific pathways, we also observed an enrichment in the GO terms related to the MAPK/ERK cascade (Fig. 3A), known to be one of the non-canonical pathways activated by TGFβ^37^. Thus, the GO analysis based on the cluster of up-regulated genes clearly highlighted the TGFβ and Wnt signaling pathways as major ones being affected in the cells exposed to egg extract reprogramming factors.

#### TGFβ signaling

TGFβ signaling is involved in numerous biological processes related to embryonic development. We then focused on the actors of the TGFβ pathway present in our goldfish DEG list (irrespective of their up or down regulation). TGFβ belongs to the superfamily of the growth factors, divided into several subfamilies including TGFβs, and Bone Morphogenetic Proteins (BMPs). For TGFβ signal to be transduced, the TGFβ ligand binds type II receptors. Ligand—type II receptor complex triggers the recruitment of TGFβ type I receptor, and the dimerized receptors subsequently activates specific Smad proteins, able to induce transcription of the TGFβ target genes^38,39^. Beyond signaling pathways involving Smads, known as canonical TGFβ pathways, other pathways independent of Smads are also controlled by TGFβ, including the MAPK Erk1/ERk2 pathway identified above by GO analysis (Fig. 3A). We therefore analyzed the expression profile of these TGFβ actors and their biological partners.

**Figure 4:**
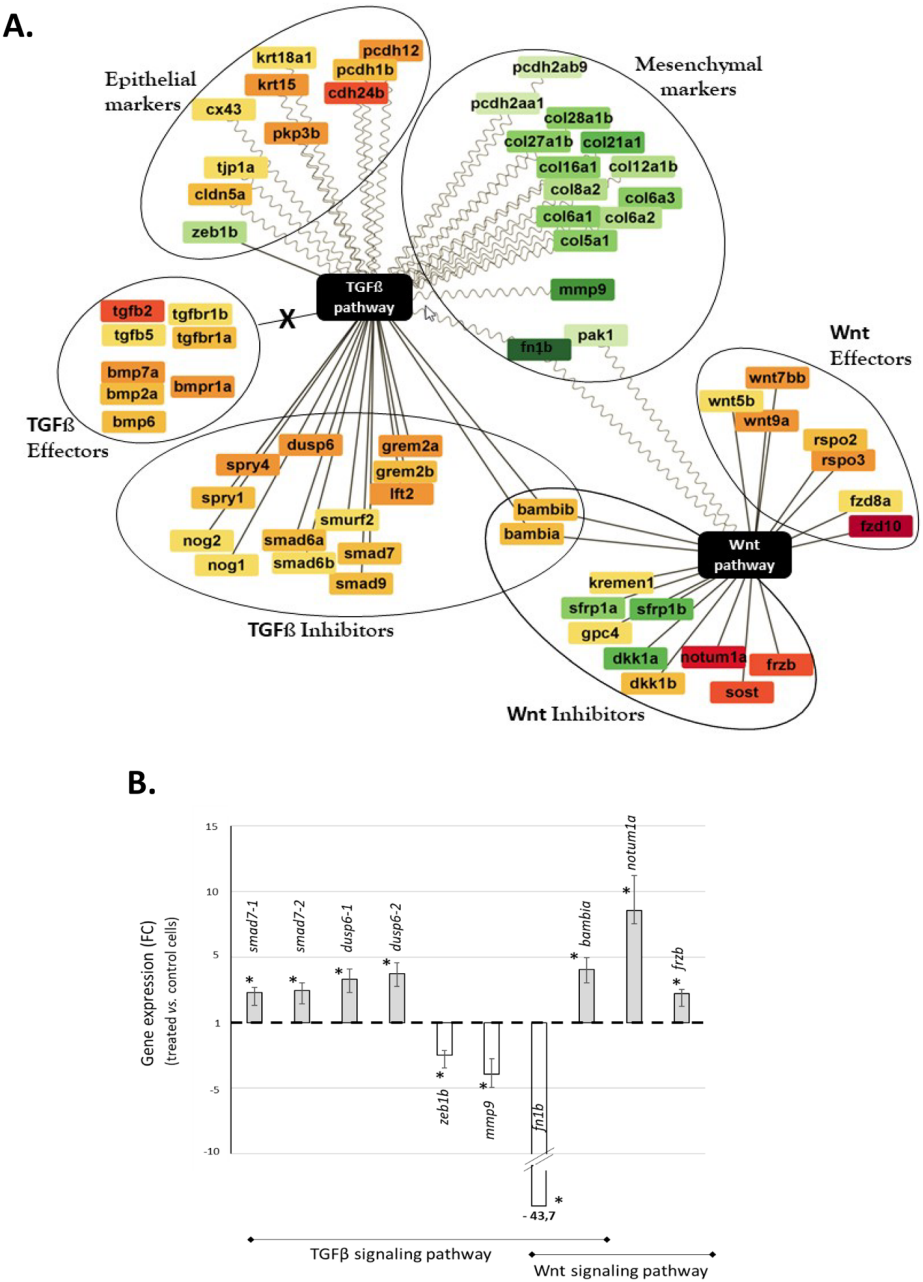
Differentially expressed genes related to TGFβ and Wnt signaling pathways. (**A**) Cytoscape^76^ representation of the genes described in Tables 1, 2. The darkest the node color, the highest the fold change (down regulation in green shades, upregulation in red shades; see Tables 1, 2 for fold change values). (**B**) Expression profile from qRT-PCR analysis of several genes associated to the TGFβ signaling pathway (*smad7-1, smad7-2, dusp6-1, dusp6-2, zeb1b, mmp9*), to Wnt/β-catenin signaling pathway (*notum1a, frzb*), to both pathways (*bambia, fn1b*), a mesenchymal marker gene (*col1a1a*), and a set of genes related to pluripotency (*nanog, pou2, sox2, c-myca1 and c-myca2*). Data are presented as fold change (FC) between treated and non-treated (control) cells. Error bars: SEM errors of the FC mean. A total of 5 to 9 paired samples (treated versus control cells) were analyzed per gene. Statistical test was performed using the nonparametric Wilcoxon test comparing the paired normalized expression values between egg extract treated and control cells. *Significant differences (p < 0.05) between treated and control values. Grey bars: upregulated genes; white bars: down regulated genes. Extensions -1 or -2 in the gene name correspond to duplicated gene copies in goldfish. It should be noted that these qPCR data confirm the microarray results indicating a deregulation of both signaling pathways.

We found that some TGFβ and BMP ligands together with type I receptors were upregulated in treated cells compared to controls (Table 1 and Fig. 4A, TGFβ Effectors). However, TGFβ type II receptors required to mediate the signal did not change their expression pattern in treated cells. Besides, the expression of the inhibitors acting upstream of the TGFβ signaling were upregulated in the treated cells (Table 1 and Fig. 4A, TGFβ Inhibitors). Among them, we identified extracellular inhibitors (*lft2, nog1, nog2, grem2a, grem2b*) and membrane inhibitors (b*ambia* and b*ambib*) that are binding to TGFβ and BMP ligands. Such binding prevents TGFβ and BMP to attach to their own receptors, thereby preventing signal transduction activity^40,41^. Beyond these inhibitors, we also found intracellular inhibitors (involved in TGFβ canonical signaling pathway) which included specific Smads (*smad6a, smad6b, smad7, smad9*) and the ubiquitin ligase *smurf2* (Table 1 and Fig. 4A,B). The combined action of Smad7 and Smurf2 is known to induce TGFβ type I receptor degradation by the proteasome^39,42^, leading to the inhibition of the TGFβ canonical pathway. Finally, *spry1, sry4,* and *dusp6* genes, inhibiting the MAPK/ERK pathway (non-canonical TGFβ pathway), were also found upregulated in the treated cells (Table 1 and Fig. 4A).

**Table 1:** List of the TGFβ signaling actors that were upregulated in treated cells.

		Gene Symbol Danio rerio	Fold Change (treated *vs.* control)	Location	Description
TGFß Effectors	TGF-ß ligands	*tgfb2*	6,7	Extra-cellular	Transforming growth factor, beta 2 [ZFIN; Acc:ZDB-GENE-030723-3]
		*tgfb5*	2,7	Extra-cellular	Transforming growth factor, beta 5 [ZFIN; Acc:ZDB-GENE-130425-3]
	TGF-ß receptors	*tgfbr1a*	2,8/3,5	Membrane	Transforming growth factor, beta receptor 1 a [ZFIN; Acc:ZDB-GENE-051120-75]
		*tgfbr1b*	2,2/2,2/2	Membrane	Transforming growth factor, beta receptor 1 b [ZFIN; Acc:ZDB-GENE-091027-1]
	BMP ligands	*bmp2a*	3,2/2,8/2,8/2,8	Extra-cellular	Bone morphogenetic protein 2a [ZFIN; Acc:ZDB-GENE-980526-388]
		*bmp6*	2,8/3,1	Extra-cellular	Bone morphogenetic protein 6 [ZFIN; Acc:ZDB-GENE-050306-42]
		*bmp7a*	5,4	Extra-cellular	Bone morphogenetic protein 7a [ZFIN; Acc:ZDB-GENE-000208-25]
	BMP receptor	*bmpr1a*	4,4	Membrane	Bone morphogenetic protein receptor, type IBa [NCBI gene; Acc:30742]
TGFß Inhibitors	Upstream of signaling pathway	*bambia*	3,2/2,7	Membrane	BMP and activin membrane-bound inhibitor (Xenopus laevis) homolog a [ZFIN; Acc:ZDB-GENE-010416-1]
		*bambib*	3,1	Membrane	BMP and activin membrane-bound inhibitor homolog (Xenopus laevis) b [ZFIN; Acc:ZDB-GENE-040704-30]
		*lft2*	4,9	Extra-cellular	lefty2 [ZFIN; Acc:ZDB-GENE-990630-11]
		*grem2a*	4,2/4,1	Extra-cellular	Gremlin 2, DAN family BMP antagonist a [ZFIN; Acc:ZDB-GENE-131127-498]
		*grem2b*	3,5	Extra-cellular	Gremlin 2, DAN family BMP antagonist b [ZFIN; Acc:ZDB-GENE-030911-9]
		*nog1*	2,6/2,6/2,4	Extra-cellular	Noggin 1 [NCBI gene; Acc:30174]
		*nog2*	2,3	Extra-cellular	Noggin 2 [NCBI gene; Acc:30185]
	Canonical signaling pathway	*smad6a*	4, 1/2,8	Intra-cellular	SMAD family member 6a [ZFIN; Acc:ZDB-GENE-011015-1]
		*smad6b*	2,3/2,2	Intra-cellular	SMAD family member 6b [ZFIN; Acc:ZDB-GENE-050419-198]
		*smad7*	2,9/2,9/2,8	Intra-cellular	SMAD family member 7 [ZFIN; Acc:ZDB-GENE-030128-3]
		*smad9*	2,9/2,4	Intra-cellular	SMAD family member 9 [ZFIN; Acc:ZDB-GENE-031014-1]
		*smurf2*	2,1	Intra-cellular	SMAD specific E3 ubiquitin protein ligase 2 [NCBI gene; Acc:563633]
	Non canonical signaling pathway	*spry1*	3,2	Intra-cellular	Sprouty homolog 1, antagonist of FGF signaling (Drosophila) [ZFIN; Acc:ZDB-GENE-081215-2]
		*spry4*	4,5/3,4	Intra-cellular	Sprouty homolog 4 (Drosophila) [ZFIN; Acc:ZDB-GENE-010803-2]
		*dusp6*	5,8/5,6/4,2	Intra-cellular	Dual specificity phosphatase 6 [ZFIN; Acc:ZDB-GENE-030613-1]

Despite upregulation of some TGFβ effectors, upregulation of many inhibitors acting upstream and downstream of the signaling pathway are signing TGFβ inhibition in treated cells. Both canonical and non-canonical signaling pathways were affected. For each *Danio rerio* gene symbol, fold change values are given for all the corresponding isoforms found in goldfish.

#### Mesenchymal and epithelial markers expression

We found that the expression of many mesenchymal and epithelial marker genes was affected by the treatment. Among the mesenchymal markers, several members of the collagen family, matrix-metallo protease (*mmp9*) and fibronectin (*fn1b*) were downregulated (Table 2 and Fig. 4A), the *fn1b* gene being the most strongly affected (−44 fold change). The concomitant upregulation of several epithelial marker genes included cadherins (*pcdh1 cadherin-like 1, pcdh12, cdh18, cdh24b*), cytokeratins (*krt15, krt18*), and cell junction actors such as *pkp3b, cldn5a, tjp1a and cx43* (Table 2 and Fig. 4A). However, we observed from this DEG analysis and from qPCR confirmation (Fig. 4B) that one abundant mesenchymal marker, *col1a1a*, remained highly expressed in treated cells and was not differentially expressed between the two conditions (relative expression 125.0 ± 52.8 in treated cells, n = 7 ; 123.1 ± 25.8 in control cells, n = 8).

**Table 2:** List of the TGFβ target genes related to mesenchymal-epithelial transition (MET).

		Gene Symbol *Danio rerio*	Fold Change (Treated *vs. C*ontrol)	Direction of Regulation	Description
	Transcription Factor	*zeb1b*	2,2/3,3	Down	Zinc finger E-box binding homeobox 1b [ZFIN; Acc:ZDB-GENE-010621-1]
TGF-ß target genes	Mesenchymal marker genes	*fn1b*	43,7	Down	Fibronectin 1b [ZFIN; Acc:ZDB-GENE-030131-6545]
		*col5a1*	2,1/3,9/4/4,1/3,4/2,3	Down	Procollagen, type V, alpha 1 [ZFIN; Acc:ZDB-GENE-041105-6]
		*col6a1*	4,2/3,2	Down	Collagen, type VI, alpha 1 [ZFIN; Acc:ZDB-GENE-070501-6]
		*col6a2*	3,1/3,2/3,1	Down	Collagen, type VI, alpha 2 [ZFIN; Acc:ZDB-GENE-070501-7]
		*col6a3*	3,9/3,6/3,4/3,3/2,9	Down	Collagen, type VI, alpha 3 [ZFIN; Acc:ZDB-GENE-070501-8]
		*col8a2*	2,7/2,9	Down	Collagen, type VIII, alpha 2 [ZFIN; Acc:ZDB-GENE-060503-488]
		*col12a1b*	2,8	Down	Collagen, type XII, alpha 1b [ZFIN; Acc:ZDB-GENE-120215-116]
		*col16a1*	4,6/6	Down	Collagen, type XVI, alpha 1 [ZFIN; Acc:ZDB-GENE-060503-351]
		*col21a1*	9,1/6,2	Down	Collagen, type XXI, alpha 1 [ZFIN; Acc:ZDB-GENE-110607-3]
		*col27a1b*	4,4/3,9	Down	Collagen, type XXVII, alpha 1b [NCBI gene; Acc:560145]
		*col28a1b*	3,4	Down	Collagen, type XXVIII, alpha 1b [ZFIN; Acc:ZDB-GENE-160503-1]
		*pcdh2aa1*	2,1	Down	Protocadherin 2 alpha a 1 [ZFIN; Acc:ZDB-GENE-041118-13]
		*pcdh2ab9*	2,03/ 2,1/ 2,1/2,3/2,4/3,1	Down	Protocadherin 2 alpha b 9 [ZFIN; Acc:ZDB-GENE-041118-8]
		*mmp9*	12	Down	Matrix metallopeptidase 9 [ZFIN; Acc:ZDB-GENE-040426-2132]
	Epithelial marker genes	*pcdh1b*	2,3/3,3	Up	Protocadherin 1b [ZFIN; Acc:ZDB-GENE-091015-2]
		*pcdh12*	4,8	Up	Protocadherin 12 [ZFIN; Acc:ZDB-GENE-140106-126]
		*cdh24b*	8,8	Up	Cadherin 24, type 2b [ZFIN; Acc:ZDB-GENE-081104-50]
		*krt15*	5,8	Up	Keratin 15 [ZFIN; Acc:ZDB-GENE-040426-2931]
		*krt18a1*	2,4	Up	Keratin 18a, tandem duplicate 1 [NCBI gene; Acc:352912]
		*pkp3b*	4,1	Up	Plakophilin 3b [ZFIN; Acc:ZDB-GENE-130530-870]
		*cldn5a*	3,4	Up	Claudin 5a [ZFIN; Acc:ZDB-GENE-040426-2442]
		*tjp1a*	2	Up	Tight junction protein 1a [ZFIN; Acc:ZDB-GENE-031001-2]
		*cx43*	2,1	Up	Connexin 43 [NCBI gene; Acc:30236]

Treated cells were characterized by the downregulation of several mesenchymal markers, especially fn1b and collagens, and by the upregulation of several epithelial markers. For each *Danio rerio* gene symbol, fold change values are given for all the corresponding isoforms found in goldfish.

#### Wnt signaling

The second signaling pathway whose terms were enriched in the GO analysis is the Wnt signaling pathway, and particularly the canonical one (Fig. 3A). Because β-catenin is a key effector of Wnt signaling, the canonical pathway is referred to as Wnt/β-catenin signaling. The transduction of Wnt signal requires Wnt-induced activation of the receptors complex made of Frizzled *(*Fzd*)* and low-density lipoprotein co-receptor related 5 or 6 (Lrp5/6). In other words, binding of the Wnt ligand to both receptors (Fzd and Lrp5/6) creates and activates the receptors complex. This initiates a series of molecular events that will protect cytosolic β-catenin from degradation. After nuclear import, β-catenin subsequently triggers the transcription of Wnt target genes by binding to transcription factors belonging to the T-cell factor/Lymphoid enhancer factor (Tcf/Lef) family^43–45^.

Our gene to gene analysis of these Wnt-related actors revealed a strong deregulation of the Wnt/β-catenin signaling pathway in egg-extract treated cells (Table 3 and Fig. 4A) with Wnt effectors being upregulated while inhibitors were down- and upregulated. Upregulated transcripts of Wnt effectors included some secreted Wnt ligands and the Fzd receptors, the expression of *fzd10* being especially strong. Moreover, transcripts of the extracellular Wnt agonists R-spondins (*rspo2, rspo3*), known to increase Fzd receptors availability on the cell surface^43^ and to stabilize the Lrp5/6 co-receptors^44^, were up regulated in treated cells. Additionally, down regulation of the extracellular inhibitors transcripts *sfrp1a, sfrp2* and *dkk1a*^43^ should be inducing a better availability of the Wnt ligand for fzd receptors. However, expression of the the co-receptors *lrp5/6* gene whose protein is necessary to activate the receptors complex was not changed by the treatment. Besides, the transcripts of many extracellular inhibitors upstream of the signaling pathway were upregulated in treated cells (Table 3). These included (i) *notum1a* and *frzb* whose proteins are known to prevent Wnt ligand from binding to Fzd receptor^44,46^, (ii) *sclerostin* (*sost*) and *dkk1b*, whose proteins are blocking Wnt-Fzd-Lrp5/6 complex formation by interacting with Lrp5/6^43^ and, (iii) *kremen1*, the gene of a membrane receptor which interacts with Dkk1 to increase the removal of the Lrp5/6 co-receptors from the cell surface by endocytosis^47^. Finally, the last interesting actors concerns the Tcf/Lef transcription factors, known as *Tcf1, Tcf3, Tcf4* and *Lef1* genes in mammals, which can either activate the Wnt target genes (when bound to to β-catenin) or repress them (when β-catenin is not available)^45^. In our study, *tcf7* (orthologue of *Tcf1* in mice) expression was upregulated in treated cells while two target genes of the Wnt pathway, the senescence gene *pak1*, also known as *p21,* and the mesenchymal marker *fn1b,* were downregulated (Table 3).

**Table 3:** List of the Wnt/β-catenin signaling actors that were differentially expressed between treated and control cells.

		Gene Symbol *Danio rerio*	Fold Change (Treated *vs. C*ontrol)	Direction of Regulation	Location	Description
Wnt effectors	Ligands	*wnt5b*	2,1	Up	Extra-cellular	Wingless-type MMTV integration site family, member 5b [NCBI gene; Acc:30105]
		*wnt7bb*	3,6/3,6/3,9/4,4	Up	Extra-cellular	Wingless-type MMTV integration site family, member 7Bb [ZFIN; Acc:ZDB-GENE-081006-1]
		*wnt9a*	3,9/4	Up	Extra-cellular	Wingless-type MMTV integration site family, member 9A [ZFIN; Acc:ZDB-GENE-060825-97]
	Receptors	*fzd8a*	2,5	Up	Membrane	Frizzled class receptor 8a [ZFIN; Acc:ZDB-GENE-000328-3]
		*fzd10*	18,1/25,9	Up	Membrane	Frizzled class receptor 10 [ZFIN; Acc:ZDB-GENE-990415-220]
	Wnt agonists	*rspo2*	3,2/3,6	Up	Extra-cellular	R-spondin 2 [ZFIN; Acc:ZDB-GENE-060503-667]
		*rspo3*	5,4/7,5	Up	Extra-cellular	R-spondin 3 [NCBI gene; Acc:100007702]
Wnt Inhibitors		*bambia*	3,2/2,7	Up	Membrane	BMP and activin membrane-bound inhibitor (Xenopus laevis) homolog a [ZFIN; Acc:ZDB-GENE-010416-1]
		*bambib*	3,1	Up	Membrane	BMP and activin membrane-bound inhibitor homolog (Xenopus laevis) b [ZFIN; Acc:ZDB-GENE-040704-30]
		*notum1a*	4/10,4	Up	Extra-cellular	Notum, palmitoleoyl-protein carboxylesterase a [NCBI gene; Acc:570510]
		*frzb*	7,15	Up	Extra-cellular	Frizzled related protein [ZFIN; Acc:ZDB-GENE-990715-1]
		*sfrp1a*	3,75	Down	Extra-cellular	Aecreted frizzled-related protein 1a [ZFIN; Acc:ZDB-GENE-040310-5]
		*sfrp1b*	7,5	Down	Extra-cellular	Secreted frizzled-related protein 2 [ZFIN; Acc:ZDB-GENE-061013-293]
		*sost*	6/7/8,7	Up	Extra-cellular	Sclerostin [NCBI gene; Acc:100000500]
		*dkk1a*	5,5/9,5	Down	Extra-cellular	Dickkopf WNT signaling pathway inhibitor 1a [ZFIN; Acc:ZDB-GENE-090313-406]
		*dkk1b*	3,1	Up	Extra-cellular	Dickkopf WNT signaling pathway inhibitor 1b [ZFIN; Acc:ZDB-GENE-990708-5]
		*kremen1*	2,1	Up	Membrane	Kringle containing transmembrane protein 1 [NCBI gene; Acc:100141352]
		*gpc4*	2,1	Up	Membrane	Glypican 4 [ZFIN; Acc:ZDB-GENE-011119-1]
Others Wnt actors	Transcription factors	*tcf7*	3,9	Up	nucleus	Transcription factor 7 [ZFIN; Acc:ZDB-GENE-050222-4]
		*tcf7l1a*	2,3/3,2	Down	nucleus	Transcription factor 7 like 1a [NCBI gene; Acc:30523]
		*tcf7l1b*	2	Down	nucleus	Transcription factor 7 like 1b [NCBI gene; Acc:30556]
	Target genes	*pak1*	2,2	Down	-	p21 protein (Cdc42/Rac)-activated kinase 1 [ZFIN; Acc:ZDB-GENE-030826-29]
		*fn1*	43,7	Down	-	Fibronectin 1b [ZFIN; Acc:ZDB-GENE-030131-6545]

For each *Danio rerio* symbol gene, fold change values (up and down) are given for all the corresponding isoforms found in goldfish. The overall disturbance of the Wnt/β-catenin signaling pathway in response to egg extract treatment, indicated by up/down regulation of effectors, inhibitors and transcription factors, tilts towards Wnt signaling inhibition.

#### Altered cell adhesion of the treated cells and genes dysregulations

In addition to the change in treated cells morphology, the highly reduced ability of the cells to adhere throughout the culture process could be due to changes in some gene expression. And indeed, from the GO analysis of all DEGs between treated and control cells, one biological process GO term highlighted the cell adhesion process (GO: 0007155; *P* value = 3.2560E−08; FDR = 1.17E−05). Furthermore, *fn1b* transcript of fibronectin (Fn1b), a major protein of the extracellular matrix which is providing highly adhesive capacity to the cells by interaction with integrin transmembrane receptors, was among the most highly downregulated genes in our conditions (Table 2).

### Some pluripotency markers remained silent in the treated cells

In order to characterize further the changes induced in the treated fin cells, we focused on some marker genes related to pluripotency, previously characterized in goldfish during early development: *pou2* (*pou5f3* in zebrafish, *oct4* in mammals), *nanog*, *sox2* and *c-myc*^32,33,48^. We observed that none of these genes were identified among the DEGs, and their expression levels remained undetectable on the microarray. These observations were confirmed by qPCR validation that *pou2*, *nanog*, *sox2* and *c-myc* expression was below detection in both treated and control cells. The DNA methylation profile of *nanog* and *pou2* promoter regions in our treated cells was also analyzed, to assess whether some DNA demethylation took place at these marker sites after *xenopus* egg treatment. This would be a necessary step in order to enable these gene transcription. Analysis of the CpG sites in *pou2* and *nanog* promoter regions revealed that they did not underwent any significant demethylation in treated cells (Supplementary Fig. S3). Although the methylation of some CpG sites was lower in treated cells compared to controls, there was no significant differences in the overall DNA methylation rate of *pou2* and *nanog* promoter regions.

### Alteration of de novo lipid biosynthesis in response to egg-extract treatment

**Figure 5:**
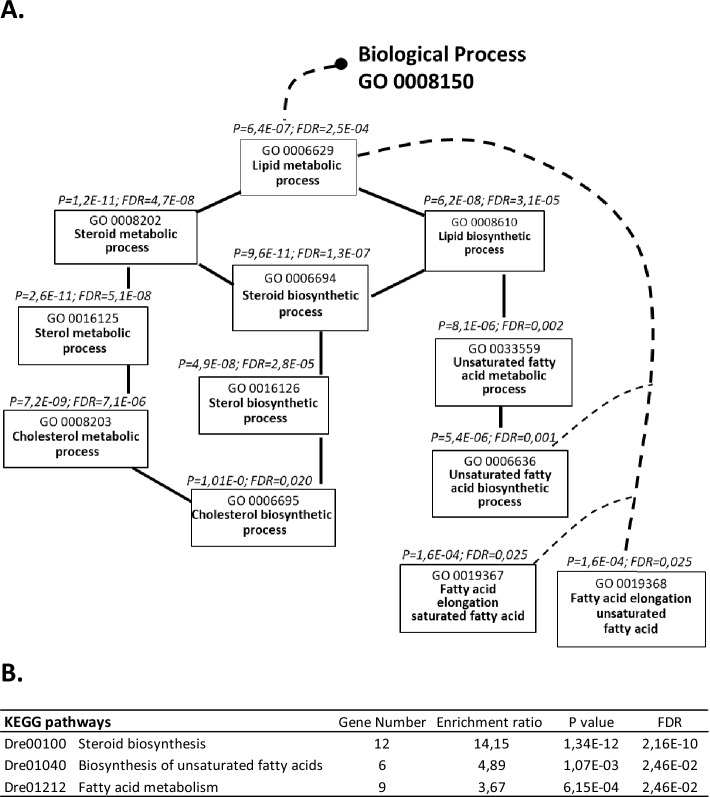
Gene Ontology (GO) flow diagram of the terms related to lipid metabolic process (**A**) and KEGG pathways (**B**). The analysis was performed on the cluster of genes downregulated in treated cells (fold change > 2) using WebGestalt web tool. The set of genes spotted on the microarray was used as the reference gene list. (**A**) The black and the dotted lines represent respectively direct and indirect connections between GO terms. (**B**) Dre, Danio rerio prefix of the KEGG identifier. For each GO term and KEGG pathway (www.kegg.jp/kegg/kegg1.html), p values (P) below 0.05 and false discovery rate (FDR) below 0.05 are indicated. Both A and B figures highlight the disturbance of lipid metabolism after egg extract treatment, and specifically cholesterol and fatty acid biosynthesis.

Regarding the cluster of downregulated genes, the GO biological processes the most significantly affected by egg-extract are related to lipid metabolism (Fig. 5A). Child GO terms targeted biosynthesis of steroid including cholesterol, and biosynthesis of unsaturated fatty acid. This was consistent with KEGG analysis showing the enrichment of the biosynthesis pathways of steroids, unsaturated fatty acids as well as the pathway of fatty acid metabolism (Fig. 5B). In this process, acetyl-CoA represents the main precursor for de novo lipid biosynthesis. Produced in the mitochondria after glycolysis, acetyl-coA has to be metabolized into citrate so that it can exit the mitochondria. Once in the cytoplasm, citrate is then converted into lipogenic acetyl-CoA (see the molecular actors of lipogenesis in^49^). A detailed analysis of lipid metabolism genes showed a downregulation of several genes involved in the cytosolic synthesis of acetyl-CoA i.e. *slc25a1b*, a key mitochondrial transporter of citrate, *aclya*, which converts cytoplasmic citrate to acetyl-CoA, *acss2*, which produces acetyl-CoA from acetate, and the acyl transferase *acat2* (Table 4). Lipid biosynthesis is also controlled by Srebf1/2 transcription factors, whose expression was downregulated in our treated cells. The target genes of these transcription factors were downregulated as well. These included transcripts of key enzymes for biosynthesis of cholesterol (*hmgcs1, hmgcra1, msmo1, fdft1, cyp51, dhcr7*) and fatty acid (*fasn, sdc, elov1a, elov2, elov5, elov6*) (Table 4). Overall, our results clearly indicate that the treated cells have strongly reduced their de novo lipid biosynthesis compared to control cells.

**Table 4:** List of the genes associated with lipid biosynthesis that were downregulated in treated cells.

	Gene Symbol *Danio rerio*	Fold Change (Treated *vs.* control)	Description
Acetyl-CoA synthesis	*slc25a1b*	6,6/11,7/14,2	slc25a1 solute carrier family 25 member 1b [NCBI gene; Acc:795332]
	*aclya*	4,4/4,5	ATP citrate lyase a [ZFIN; Acc:ZDB-GENE-031113-1]
	*acss2l*	6,63/32,6	acyl-CoA synthetase short chain family member 2 like [ZFIN; Acc:ZDB-GENE-130530-723]
	*acsl3a*	59,8	acyl-CoA synthetase long chain family member 3a [ZFIN; Acc:ZDB-GENE-050420-181]
	*acat2*	3/5	acetyl-CoA acetyltransferase 2 [ZFIN; Acc:ZDB-GENE-990714-22]
Fatty acid biosynthesis	*fasn*	2,8/2,8/3,4	fatty acid synthase [ZFIN; Acc:ZDB-GENE-030131-7802]
	*scd*	6,5/6,9/7,4	stearoyl-CoA desaturase (delta-9-desaturase) [ZFIN; Acc:ZDB-GENE-031106-3]
	*fads2*	32,2/34,8/35/35,6	fatty acid desaturase 2 [NCBI gene; Acc:140615]
	*elov1a*	2,2/2,4	ELOVL fatty acid elongase 1a [ZFIN; Acc:ZDB-GENE-041010-66]
	*elov2*	3/22,3	ELOVL fatty acid elongase 2 [ZFIN; Acc:ZDB-GENE-060421-5612]
	*elov5*	2,8/5,5	ELOVL fatty acid elongase 5 [ZFIN; Acc:ZDB-GENE-040407-2]
	*elov6*	2,1/9,6	ELOVL fatty acid elongase 6 [NCBI gene; Acc:317738]
Cholesterol biosynthesis	*hmgcs1*	13,2/12,4	3-hydroxy-3-methylglutaryl-CoA synthase 1 (soluble) [ZFIN; Acc:ZDB-GENE-040426-1042]
	*hmgcra*	5,1 /5,2	3-hydroxy-3-methylglutaryl-CoA reductase a [ZFIN; Acc:ZDB-GENE-040401-2]
	*msmo1*	22,6	methylsterol monooxygenase 1 [NCBI gene; Acc:406662]
	*fdft1*	6,3/6,7/6,7	farnesyl-diphosphate farnesyltransferase 1 [ZFIN; Acc:ZDB-GENE-081104-242]
	*cyp51*	10,6 /10,9	cytochrome P450, family 51 [NCBI gene; Acc:414331]
	*dhcr7*	8,4/8,8	7-dehydrocholesterol reductase [NCBI gene; Acc:378446]
	*dhcr24*	8,1	24-dehydrocholesterol reductase [ZFIN; Acc:ZDB-GENE-041212-73]
	*cyp7a1*	4,6/5,5	cytochrome P450, family 7, subfamily A, polypeptide 1 [ZFIN; Acc:ZDB-GENE-040426-1296]
	*mvda*	4,4	mevalonate (diphospho) decarboxylase a [NCBI gene; Acc:492781]
Transcription factors *	*srebf1*	3,3/4	sterol regulatory element binding transcription factor 1 [ZFIN; Acc:ZDB-GENE-090812-3]
	*srebf2*	2,6/2,8	sterol regulatory element binding transcription factor 2 [NCBI gene; Acc:100037309]

For each *Danio rerio* symbol gene, fold change values are given for all the corresponding isoforms found in goldfish. *Transcription factors involved in the regulation of fatty acid and cholesterol de novo synthesis.

## Discussion

In this work, we explored to what extent fin somatic cells in culture could be modified by exposure to *Xenopus* egg-extracts. Our objective was to induce some relaxing in the differentiated program of these cells, with the ultimate goal, not tested here, that they would be better fitted for the extensive reprogramming that has to take place after nuclear transfer in fish. We chose the heterologous *Xenopus* egg extract as reprogramming trigger, and not goldfish one. Indeed, because of their meroblastic cleavage, goldfish oocytes contain very little cytoplasm^31^, and our preliminary testing had shown overabundant vitellogenin in the extracts (Supplementary Figure S4.). This was jeopardizing the chances to have cells truly exposed to oocyte cytoplasmic factors. Moreover, no specific antibodies were available to validate the stage of the goldfish oocyte extracts (MII), so we had no tools to assess its quality. Last, *Xenopus* egg extract have been shown to induce some changes in mammalian cultured cells, so it was known that they had the potential to be efficient in distant species. Besides, we observed that they contain much more cytoplasmic factors than did goldfish oocyte extracts.

We showed that the treatment with *Xenopus* egg extract triggered some phenotypic changes of the cells, namely reduced adhesion capacity, adoption of a cubic shape morphology (epithelial-like), and inability to survive in L15 medium. This is why a medium more fit for these modified cells had to be devised. In mammals, somatic cells treated with egg extract were reported to be cultured in embryonic stem (ES) cells medium containing Leukemia Inhibitory Factor (LIF) and other complements aimed to prevent cell differentiation^24,26,28^. However, fish ES-like cells were known to be independent from LIF (reviewed in^50^). Furthermore, the maintenance of an undifferentiated state in zebrafish and medaka ES-like cultured cells was reported to require a medium enriched with fish serum and species-specific embryo extracts^50,51^. This led us to devise a specific ESM4 medium in which the embryo extracts were obtained from goldfish embryos, and this medium proved to be better suited to sustain survival and proliferation of the treated cells. In addition to these phenotypic changes, transcriptomic analysis led to the identification of two clusters of differentially expressed genes. We showed by GO analysis that cell surface receptor signaling pathways and lipid metabolism were the most significant terms that stood out from the list of these genes. Actors of the TGFβ and Wnt/β-catenin signaling pathways had altered expression , and this was combined with expressional changes indicative of MET initiation. These changes were associated with the lack of restoration of pluripotent markers activity and of their promoter demethylation, and with a reduction of lipid biosynthesis.

### Significance of the expressional changes observed in the cells treated by egg extract

It is well described that reprogramming of somatic cells into a less differentiated state, be it after induced pluripotency, nuclear transfer, or cell fusion, encompasses a series of molecular changes whose sequence includes the downregulation of somatic markers and of some signaling networks, the induction of MET, and the activation of early pluripotency markers^52,53^. However, how egg extract treatment of somatic cultured cells would affect their differentiated program had never been explored in fish, and we had no preconceived reprogramming target except for the expression of few marker genes already studied in mammals (including *Oct 4* and *Nanog*). The unsuitability of the L15 medium and reliance of our treated cells on the ESM4 medium for survival and proliferation provided a first indication that the egg extract treatment was inducing some changes in the treated cell physiology. Our microarray analysis provided a much more comprehensive view of the molecular changes at stake. The expressional changes observed with the TGFβ signaling actors in our treated cells tilted the balance in favor of an overall inhibition of the TGFβ signaling pathway. Indeed, as summarized Fig. 4A, we showed that numerous inhibitors of this pathway including those of the MAPK/ERK pathway (non-canonical TGFβ pathway) were upregulated after the treatment. Besides, although some TGFβ effectors were upregulated, they missed the concomitant upregulation of an essential mediator of their action, that is the TGFβ type II receptors, whose expression was unchanged. This TGFβ signaling inhibition would be one preliminary step in the cellular reprogramming process. Indeed, experimental inhibition of TGFβ signaling was shown to cooperate in the reprogramming of murine fibroblasts into iPSCs^54–56^. Furthermore, ERK inhibition was also shown to be an early molecular signature of somatic cell reprogramming in this model species^57^, and inhibition of both TGFβ receptors and ERK^58^ also improved fibroblast reprogramming^59^. In mammals, one consequence of TGFβ signaling inhibition is the induction of MET, considered to be a hallmark of iPSC early phase reprogramming, and described as crucial for reaching pluripotency^54,55,58,60,61^. MET is characterized by the loss of mesenchymal markers and by the activation of genes determining epithelial fate^60^. The upregulation observed in our study for the expression of epithelial markers, and the downregulation of the mesenchymal markers, strongly suggest the initiation of a MET program in the treated cells. For example, the gap junction gene *cx43* upregulated in our conditions is known to be specifically enriched in epithelial cells and iPSCs, and its ectopic expression and gene upregulation has been associated with an increase in reprogramming efficiency by facilitating mesenchymal-epithelial transition (MET)^62^. Pak1 (down regulated in this study) is a senescence actor shown to be a barrier to iPSCs reprograming^63,64^ and to MET. We therefore infer that its inhibition was favorable to MET and reprogramming in our treated cells. Also, the transcription factor gene *zeb1b* downregulated in our conditions is known to induce EMT (epithelial-mesenchymal transition)^65^, ie the reverse of the MET. These altered expressional profiles indicative of the triggering of an epithelial program are in accordance with the epithelial-like morphology observed on the treated cells, which were more cubic than the elongated control cells. However, maintenance of high expressional levels of the mesenchymal *col1a1a* suggests that MET would be initiated but not terminated in our culture conditions. Taken as a whole, our results lead us to propose that the egg extract would have initiated a reprogramming of the fin cells by directing them towards a MET via inhibition of TGFβ signaling.

The status of the changes observed on the Wnt signaling pathway actors is more complex to settle. From one side, the observed increase in Wnt ligands and *fzd* receptors gene expression should have favored the stimulation of the Wnt pathway, but expression of *lpr5/6* remained unchanged after the treatment. Therefore, such *lpr5/6* stability should stoichiometrically hamper the formation of the ternary proteic complex Wnt ligand/fzd receptor/LRP5/6 co-receptors that is essential for signal transduction of the Wnt pathway. Additionally, the fact that inhibitors upstream of this pathway were strongly upregulated (summarized Fig. 4A) would be favorable to the hypothesis of Wnt pathway inhibition. This is sustained by acute downregulation of two target genes of this pathway (*pak1* and *fn1b*), likely thanks to *Tcf7* upregulation. Such pattern would thus indicate that β catenin was not available for target gene activation by the complex Tcf7/β catenin^45^, and then that the pathway was in a downregulated state. All these observations give ground to the hypothesis that the deregulation of the Wnt signaling in our treated cells would be rather in an “off” configuration. Despite its oscillatory pattern during reprogramming, such likely Wnt off state matches the early phase of iPSCs reprogramming of mice embryonic fibroblast^66^. The off state of this signaling pathway observed in our study would indicate that our cells are in an early stage of reprogramming.

### Incomplete reprogramming of the treated cells

The process of somatic reprogramming in iPSCs is generally encompassing two phases^67^: (i) an early or initiation phase during which the somatic cells undergo a MET, lose their mesenchymal characteristics and develop an epithelial phenotype and, (ii) a late maturation phase allowing the reactivation of the pluripotency network. As explained above, the treatment applied to our culture cells was intended to increase somatic cell plasticity towards further reprogramming such as the one required after nuclear transfer. Thus, our treatment with X*enopus* egg extracts remained within physiological limits, and it could not be expected to be as thorough as after reprogramming into iPSCs. Several indicators in our study showed that indeed, the treated fin cells were not entirely changed in their transcriptomic profile. Namely, although several collagens underwent a reduced expression in the treated cells, *col1a1a* abundantly expressed in fin cells^68^ remained highly expressed after cell treatment. Furthermore, we failed to detect any re-expression of the canonical pluripotency markers that are *pou2, sox2, nanog* and *c-myc*. This is at odd with the Oct4 re-expression induced with a similar treatment in porcine or human cultured cells^25,27^. Because of the stochastic re-expression of these genes described by these authors and reviewed in^69^, we infer that we asses these markers expression in another reprogramming window, or that our cells may still be in the initiation or intermediate phase of reprogramming. It was shown previously in goldfish that *nanog* and *pou2* silenced status in fin cells is associated with the hypermethylation of a CpGs locus in their promoter region^32,33^. We also showed recently that after nuclear transfer with non-treated fin cells, these loci underwent a partial and stochastic demethylation in the developing clones^16^. This prompted us to analyze whether some DNA demethylation took place at these marker sites after *xenopus* egg treatment, as these DNA methylated sites might be more labile upon reprogramming. The silenced status of pluripotency marker genes associated with the absence of significant DNA methylation remodeling support the idea that treated cells would have been only partially reprogrammed by *Xenopus* egg-extract treatment. Our cells would not have reached the maturation phase of reprogramming characterized by Oct4 or Nanog and Sox2 re-expression as observed in mammalian somatic cells.

### Reduced lipid metabolism in the treated cells

We showed that the whole cluster of downregulated genes induced the high significance of GO terms related to lipid metabolism, and numerous actors of the lipid biosynthesis were downregulated after *Xenopus* egg treatment. This questions the role of lipids in our cellular reprogramming scheme. Indeed, studies on iPSCs indicate, on the contrary, that an increased lipid biosynthesis is favorable to MET and reprogramming^70,71^, and that conversely, inhibition of fatty acid biosynthesis blocks mouse embryonic fibroblast reprogramming to iPSCs^72^. It was shown that large amounts of lipids are consumed during the reprogramming process, as judged by the decreasing number of lipid droplets per cell between the early and late stages of reprogramming^72^. This would indicate that lipid biosynthesis upregulation is intended to provide for additional energetic resources to the cells undergoing reprogramming. This hypothesis was explored in porcine iPSCs^73^, and it was demonstrated that supplementation of the culture medium with triglycerides, free fatty-acids, phospholipids and cholesterol improved the reprogramming of embryonic fibroblasts by promoting MET. Our observed downregulation of these actors unambiguously showed that egg-extract treatment failed to remodel the lipid metabolism of fin cells according to iPSC pattern. We infer that lipid biosynthesis downregulation was a response of the treated cells to the enriched ESM4 culture medium. In the conventional L15 medium, the treated cells died after a few days. It means that if the treated cells suffered endogenous lipid exhaustion, the lipids provided by their short-term exposure to *Xenopus* egg extracts were not able to compensate for such losses. On the contrary, subsequent culture in the ESM4 medium containing extracts from goldfish embryos at 55 h post fertilization stage may have provided for the required energetic substrates. Fish embryos at this stage are indeed highly enriched in cholesterol, phosphatidyl choline and triglycerides^74^. This means that ESM4 may have provided the same MET-favorable environment as the one tested in^73^. In all, the exogenous lipid supply via ESM4 would have met the need of the treated cells, possibly supporting the MET requirements, and as a response, the lipid anabolism of the cells was reduced, leading to the observed downregulation of the corresponding genes.

## Conclusion

The treatment of fish fin cells with *Xenopus* egg extract and subsequent culture in ESM4 induced phenotypical and expressional changes. The transcriptomic approach allowed the identification without a priori of numerous actors known to be involved in cellular reprogramming and MET. The identified reprogramming markers encompassed the TGFβ and Wnt/β-catenin signaling pathways alteration, most likely inhibition, and expressional changes of genes relevant with the cubic cell shape (epithelial features) and the lessened cell adhesion capacity acquired by the treated cells. We also provided evidences that if a reprogramming was engaged, it was obviously incomplete, as attested by the lack of pluripotency markers re-expression, and maintenance of one abundant mesenchymal marker. Taken as a whole, it appears that the fish somatic cells would have acquired some markers of early reprogramming phases, indicating that the treatment helped to release some of the reprogramming barriers present in our differentiated fin cells. The observed changes could be a first favorable step when the cells are to be used for nuclear transfer, before further expressional reprogramming and chromatin remodeling are triggered at the onset of embryo development.

## Methods

### Animals

For caudal fin collection, two-year-old goldfish (*Carassius auratus*) (n = 42 females, 60 g mean weight), were obtained from outdoor ponds at INRAE U3E experimental facility (Rennes, France). They were kept in 100 L tanks with recycled water at 14 °C for several weeks at ISC INRAE LPGP (Rennes, France, agreement number D-35-238-6). Fish were euthanized by decapitation after lethal anesthesia in Tricaine (MS-222) 200 mg/mL in tank water (10 min), and caudal fins were collected on the euthanized fish. The procedure was approved by the local institutional ethic committee ‘’Structure of control for Animal Welfare in Research” (SBEA) at the Fish Physiology and Genomics department of INRAE (National Institute for Agronomic and Environmental Research) under the reference C-2019–01-CL. It complied with the French animal welfare guidelines and under the French registration authorization n° 78–25 (N. Chênais).

For collection of eggs, 2 years-old *Xenopus laevis* females (n = 7, 100 g mean weight) were reared in 300 L tanks in recycled water at 22 °C, at the CRB Xenope facility (University of Rennes 1, France, agreement number: 35–238‐42). The females were primed by peritoneal injection of 750 U hCG 3 days before egg collection, and stimulated with 30 U hCG 18 h before egg collection. Unfertilized eggs were obtained from natural spawning of the females kept overnight in 30 L tanks at 22 °C. This procedure was approved by the Rennes local Ethics Committee in Animal Experimentation n°07 (BBEA, Université de Rennes 1). Xenopus manipulation was performed in compliance with the French animal welfare guidelines and under the French registration authorization n° 78–25 (N. Chênais).

The animal study is reported in accordance with ARRIVE guidelines (https://arriveguidelines.org) for animal research.

### Goldfish fin cell preparation

Fin cells were isolated and cultured according to^30^. Briefly, fins were minced and digested with 2 mg/mL collagenase. Released fin cells were plated in supplemented L15 culture medium. After 24 h, adhering epithelial cells were discarded while the supernatant, enriched with slow adhering mesenchymal cells, was collected. These cells have previously been shown to be the most suitable for nuclear transfer^1,68^. After filtration and washing, the mesenchymal cells were seeded at 0.2 × 10^6^ cells per well in 24 well plates and cultured in L15 medium for 2 days (about 80% confluence) until *Xenopus* egg treatment.

### Xenopus egg extract preparation and characterization

Egg extracts were prepared as described previously^30^. Laid eggs were crushed at 10 600 g for 20 min at 4 °C. The extract was then clarified at 10 600 g for 20 min at 4 °C. The supernatant was collected, snap frozen in liquid nitrogen and stored at -80 °C. A total of 7 individual spawns were collected, providing 7 batches of independent egg extracts with a protein concentration of 40 to 50 mg/mL and an osmolality of about 400 mOsm/kg.

Egg extract stage was characterized by western blot analysis using the mitotic markers Greatwall and Cyclin B as described in^30^. For each egg extract, one fraction was immediately denatured in Laemmli buffer at 95 °C (3 min). A second fraction was incubated at 25 °C for up to 2 h prior to denaturation, to mirror the time during which cells were treated with the egg extract. A last fraction was incubated with 0.8 mM Ca^2+^ at 25 °C for 1 h to test its responsiveness to calcium-induced activation, before it was denatured. MII status of the 7 egg extracts was determined using rabbit polyclonal *Xenopus* anti-Greatwall and anti-Cyclin B (1:1000 each) according to^45^. Immunolabelling was revealed with Uptima Uptilight HRP Chemiluminescent Substrate (Uptima-Interchim 58372B). Images were acquired with Fusion FX7 (Vilbert Lourmat).

### Somatic cell treatment and culture

Adherent mesenchymal cells were permeabilized with digitonin (30 ug/mL 2 min 4 °C) before exposure to egg extract (1 h, 25 °C), according to^30^. Cells were then incubated for 2 h in growth L15 medium supplemented with 2 mM CaCl2 (25 °C) to reseal the plasma membranes and then cultured at 25 °C in ESM4 medium^50^ (Supplementary Table S1). Culture medium was changed every 3 days. After 8 days, cultured cells were collected after trypsinization and snap-frozen in liquid nitrogen. Non-permeabilized cells were grown in L15 medium and used as controls. They followed the steps as the treated cells and were snap-frozen after 8 days.

### Microarray analysis

#### Microarray preparation and hybridization

Agilent 8 × 60 K high-density oligonucleotide microarray (GEO platform no. GPL32340) was spotted with a set of 52,362 distinct goldfish oligonucleotides. Available goldfish NCBI sequences were blasted on the zebrafish genome, generating a list of zebrafish proteins identified as ENSDARP in the Ensembl database. The official symbol of each gene, its description and its Ensembl ID, called ENSDARG, were then extracted from the ENSDARPs using the Ensembl Biomart programm.

Total DNA and RNA of the cultured cells were extracted simultaneously after cell lysis in RNAsin (1 µL) in Tri-Reagent, according the instructions for Miniprep DNA/RNA Direct-zol column extraction kit (Zymo Research, R2081). RNA labeling and hybridization were performed according to the manufacturer’s instructions (Agilent “One-Color Microarray-Based Gene Expression Analysis (Low Input Quick Amp labeling)”). For each sample, 150 ng total RNA was amplified and labeled using Cy3-CTP. Yield (> 825 ng cRNA) and specific activity (> 6 pmol of Cy3 per μg of cRNA) of the obtained Cy3-cRNA were checked on Nanodrop. Cy3-cRNA (600 ng) from each sample was fragmented, and samples were hybridized on randomly chosen sub-arrays for 17 h at 65 °C. After microarray scanning (Agilent DNA Microarray Scanner, Agilent Technologies, Massy, France), data were obtained with the Agilent Feature Extraction software (10.7.3.1) according to the appropriate GE protocol (GE1_107_Sep09) and imported into GeneSpring GX software (Agilent Technologies, Santa Clara, CA, USA) for analysis. Data were published at the NCBI’s Gene Expression Omnibus^75^ and are accessible through GEO series accession number GSE205854. Of the 16 cell samples laid on the microarray, only 12 samples passed the quality controls and were selected for analysis (n = 5 control; n = 7 treated with egg extract).

#### Differentially expressed genes identification and Gene Ontology analysis

The raw gene expression data were normalized and transformed into Log2 values using GeneSpring software (Agilent). Only genes displaying an expression value significantly higher than that of the background in at least 75% of the samples and in at least one of the two conditions were retained. Selection of differentially expressed genes relied on a Student's t-test with false discovery rate (FDR) correction and a fold change > 2 was applied. The significance level was set to FDR < 0.05 and p-value < 0.05. The DEGs were then classified according to their expression profile by unsupervised hierarchical clustering using Cluster 3.0 software and were visualized by Java TreeView software (https://bitbucket.org/TreeView3Dev/treeview3/src/master/).

A gene ontology analysis was carried out on the DEGs of each cluster using WebGestalt web tool (AnaLysis web-based GEne SeT AnaLysis toolkit). In order to highlight the GO terms related to biological process that were significantly enriched, an over-representation analysis (ORA) was carried out on the gene IDs (ENSDARG) of each cluster. For this, eachgene list of interest was compared to a background gene list corresponding to all the genes spotted on the microarray. ORA was also carried out to search for KEGG (Kyoto Encyclopedia of Genes and Genomes) pathways. The significance level was set to below an FDR 5% and a p value of 0.05.

#### RT-qPCR analysis

The expression level of several genes of interest was assessed by RT-qPCR according to^68^. This included a set of genes related to TGFβ signaling pathway (*smad7-1, smad7-2, dusp6-1, dusp6-2, zeb1b, mmp9*), to Wnt/β-catenin signaling pathway (*notum1a, frzb*), as well as 2 genes common to both pathways (*bambia, fn1b*). We also analyzed a mesenchymal marker gene (*col1a1a*) and a set of genes related to pluripotency (*nanog, pou2, sox2, c-myca1* and *c-myca2*). PCR efficiency was determined for all specific primer sets using serial dilutions of RT samples. All values ranged from 95 to 105%. Primer sequence information is provided in Supplementary Table S3. Gene expressions were analyzed on 5 to 9 paired samples (treated and control cells). Gene expression values were normalized using the endogeneous *18S rRNA* control gene and calculated according the formula: 2^−ΔCt^ with ΔCt = mean Ct (*target gene*) – mean Ct (*18S rRNA*). Fold change values between treated *and* control cells was calculated using the 2^−ΔΔCT^ formula with ΔΔCt = mean ΔCt (control cells)—mean ΔCt (treated cells). Statistical significance of gene expression studies was assessed using Wilcoxon test for paired-sample comparisons (p < 0.05) on the individual 2^−ΔCt^ values of treated and control samples.

#### DNA methylation analysis

Total extracted DNA was purified using the Genomic DNA Purification and Concentration Kit (Zymo Research, D4010) and quantified using the QubitTM dsDNA HS Assay Kit (Q32851, Invitrogen). DNA was treated with bisulfite using the EZ DNA Methylation-Gold kit (Zymo Research, D5006) and regions of interest were amplified according to^16^. Methylation status of the targeted CpGs was calculated after pyrosequencing with PyroMark Q24 ID 2.5 software (QIAGEN). Bisulfite conversion of control cytosines was above 98%.

#### Availability of data and material

The agilent 8 × 60 K high-density oligonucleotide microarray used in this study is available at https://www.ncbi.nlm.nih.gov/geo/ under the accession number GPL32340.

The microarray expression data of this study are available at https://www.ncbi.nlm.nih.gov/geo/ under the Accession Number GSE205854 (from Jan 01 2023).

## Supplementary Information

Supplementary Information 1.

Supplementary Information 2.

Supplementary Information 3.

## Data Availability

Microarray data were published at the NCBI’s Gene Expression Omnibus^75^ and are accessible through GEO series accession number GSE205854. Most other data obtained in this work were provided in the supplementary file. Any missing data or supplementary information should be asked to the corresponding authors who will answer the requests in a timely manner.
