# Supplementary material for: TGFβ inhibition and mesenchymal to epithelial transition initiation by Xenopus egg extract: first steps towards early reprogramming in fish somatic cell
Source: Sci Rep. 2023 Jun 20;13:9967. doi: 10.1038/s41598-023-36354-3 (PMC10281987; doi:10.1038/s41598-023-36354-3)
Supplement: Supplementary file 1 — Supplementary Information 1. [file 41598_2023_36354_MOESM1_ESM.docx]

Supplementary Information File

**TGFβ inhibition and mesenchymal to epithelial transition initiation by *Xenopus* egg extract: first steps towards early reprogramming in fish somatic cell.**

Nathalie Chênais^1^*, Aurelie Lecam^1^, Brigitte Guillet^2^, Jean-Jacques Lareyre^1^, Catherine Labbé^1^*

^1^ INRAE, UR1037 LPGP, Fish Physiology and Genomics, Campus de Beaulieu, F-35000 Rennes, France

^2^Université de Rennes 1, Campus de Beaulieu, F-35000 Rennes, France

***CORRESPONDING AUTHOR**

Nathalie Chênais: [nathalie.chenais@inrae.fr](mailto:nathalie.chenais@inrae.fr)

Catherine Labbé: catherine.labbe@inrae.fr


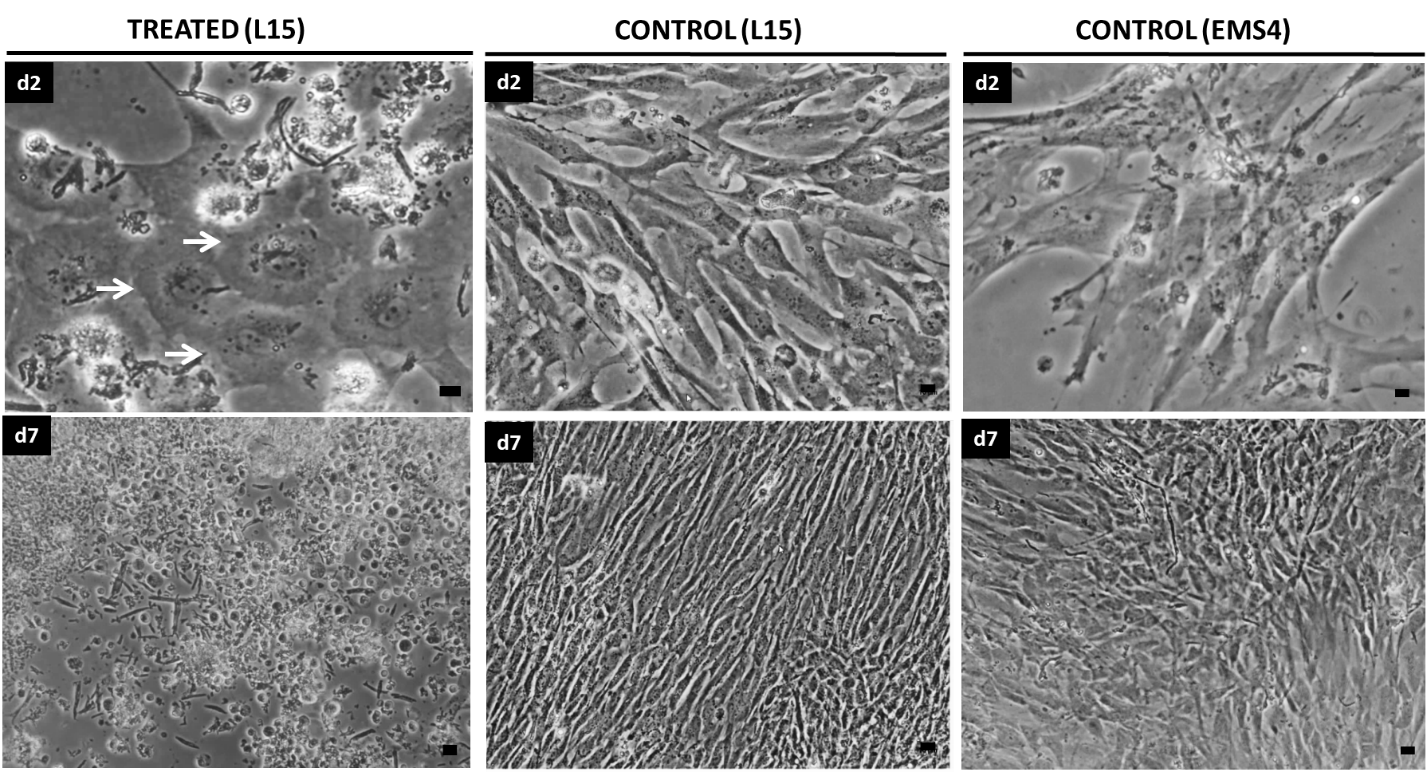


**Supplementary Fig. S1:** Morphology and fate of *Xenopus* egg extract treated fin cells over culture time in L15 medium at 25°C. Control cells (no permeabilization and no exposure to egg extract) were cultivated in L15 medium or ESM4 medium under the same conditions as the treated ones. Cells were observed by phase contrast microscopy 2 days (d2) and 7 days (d7) after the treatment. Treated cells exhibited a cubic shape at d2 post-treatment (white arrows) that is drastically different from control cells displaying an elongated shape whatever the medium (L15 or ESM4). At d7, the treated cells were unable to survive in L15 medium as shown by the presence of debris and numerous floating cells. Such observations contrast with the high density of control cells grown for 7 days in either L15 or ESM4. These pictures are representative of three experiments with different cells and egg extract batches. Scale bar = 10 µm.

| **Medium components** | **ESM4** |
| --- | --- |
| L-Glutamine | 2 mM |
| Amphotericin B | 2,5 µg/mL |
| Gentamycin | 100 µg/mL |
| Pyruvic acid | 1 % (w/v) |
| Nonessential amino acid | 1 % (w/v) |
| Sodium selenite | 2 nM |
| 2-Mercaptoethanol | 100 µM |
| Fetal bovine serum | 10 % (v/v) |
| Fish serum | 1 % (v/v) |
| Human recombinant basic FGF | 8 ng/mL |
| Goldfish embryo extract | 1 embryo/mL |

**Supplementary Table S1:** Composition of ESM4 growth medium adapted to the culture of goldfish fin cells treated with *Xenopus* egg extract. The components listed in the table were added to the basic medium L15 medium supplemented with glucose (4.5 g/L), hepes (5 mM), sodium bicarbonate (2 mM) and adjusted to pH 7.3.


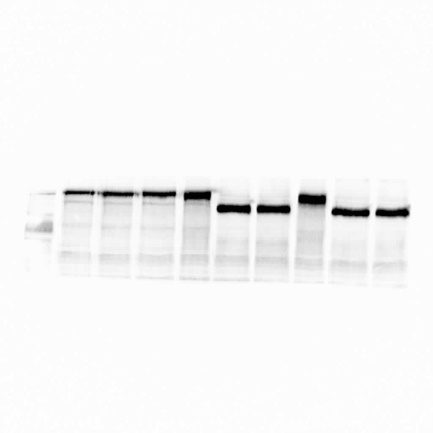

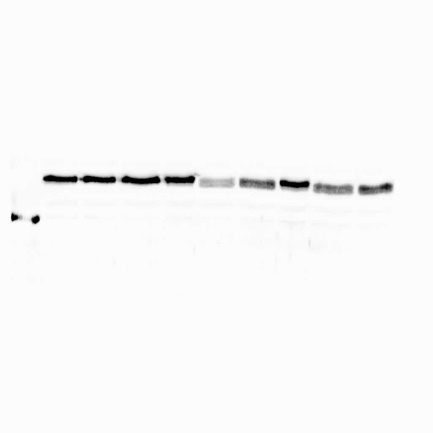

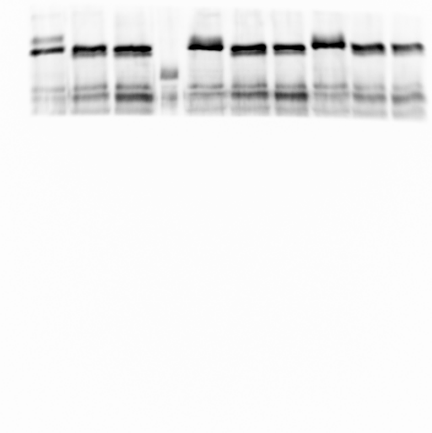

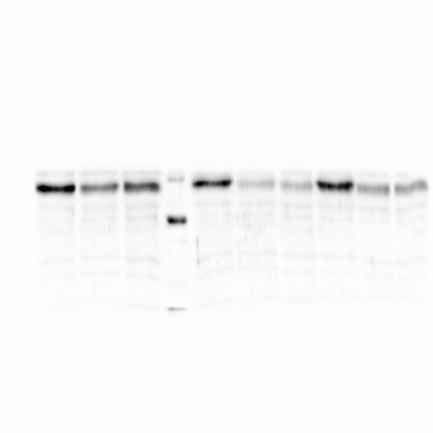


(corresponding original pictures of the original blots)

Other pictures of the MII blots, at different exposure time (top = Gwl antibodies, bottom = Cyclin B antibodies)


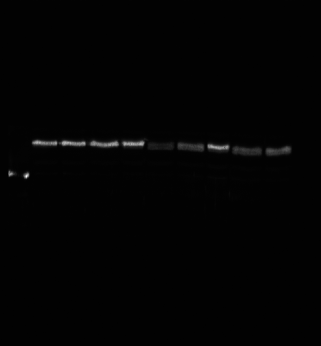

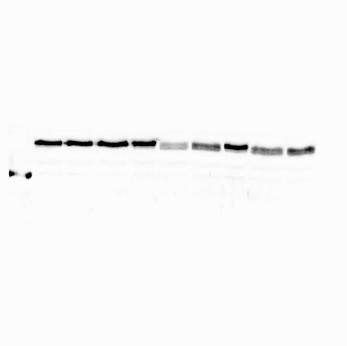

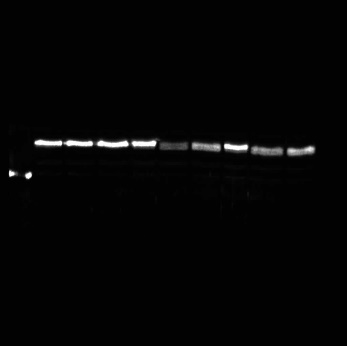

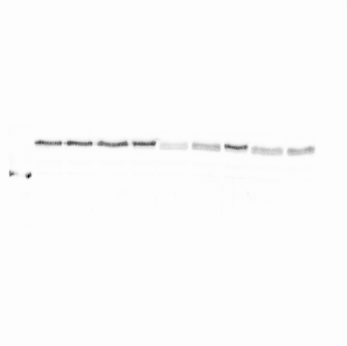

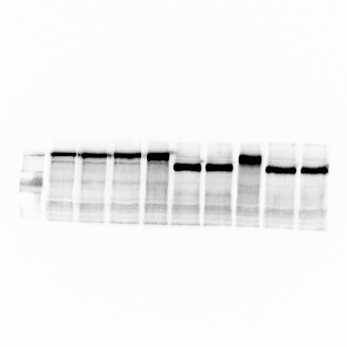

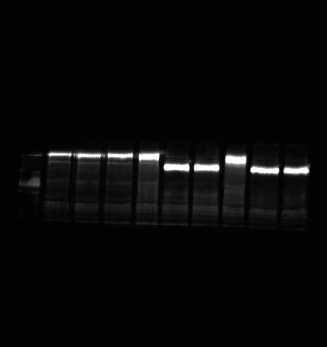

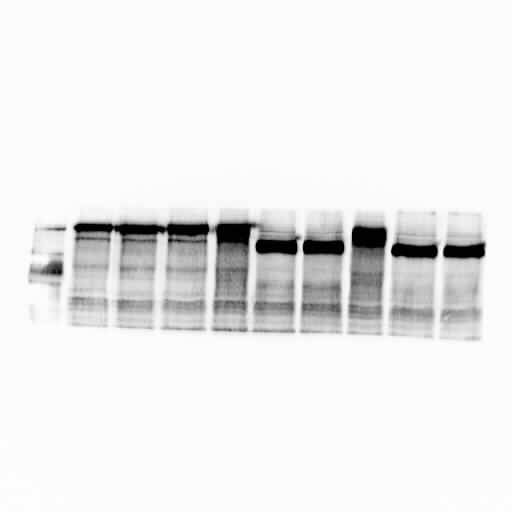

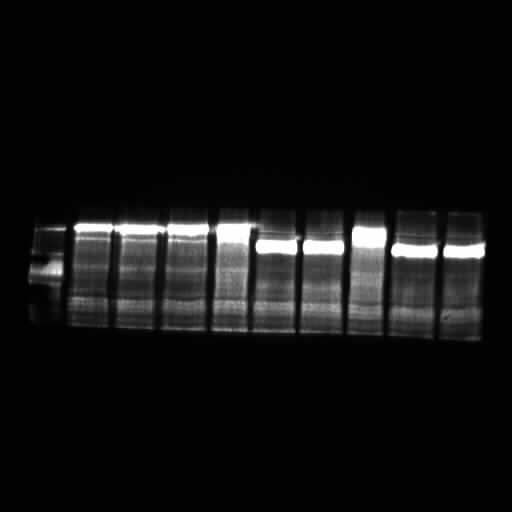


Other pictures of the MII-late blots, at different exposure time (top = Gwl antibodies, bottom = Cyclin B antibodies)


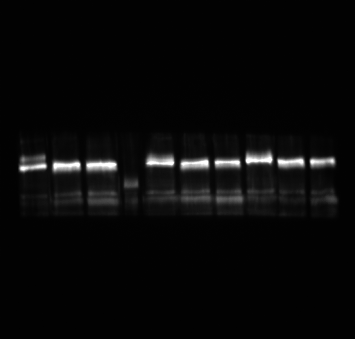

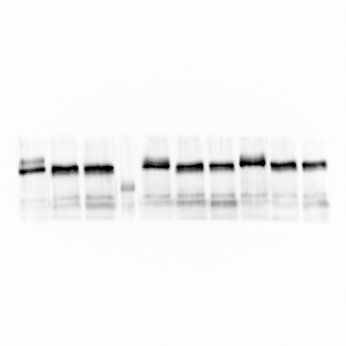

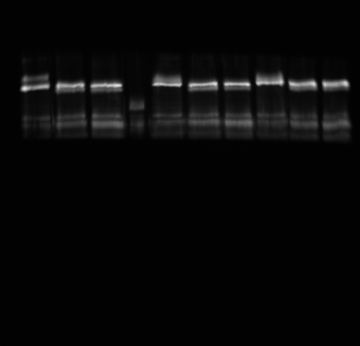

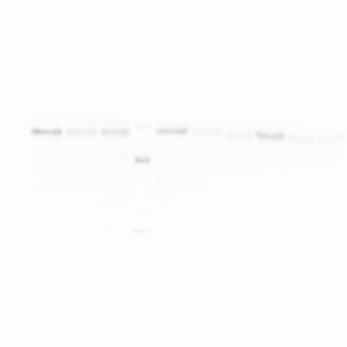

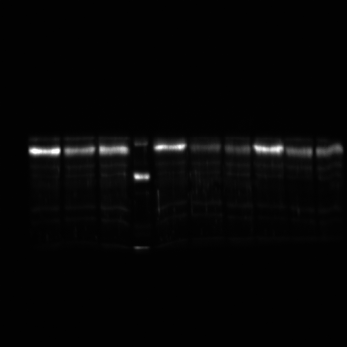

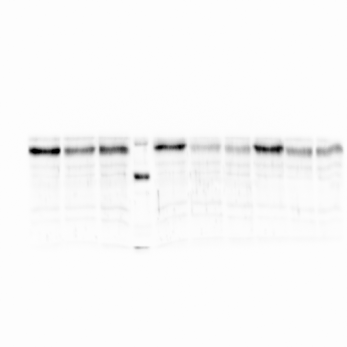

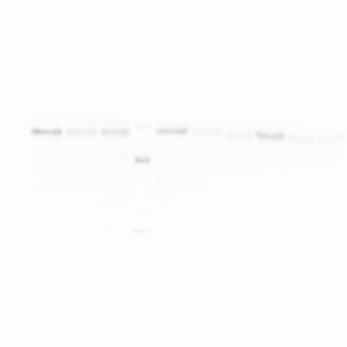

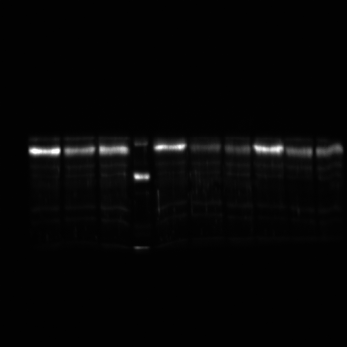

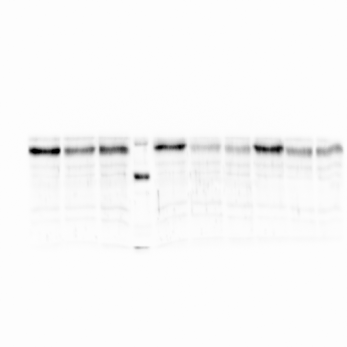


**Supplementary Fig. S2:** Mitotic status of egg extracts from *Xenopus* individual spawns: molecular characteristics of their stage (MII or MII-late) by Western blot using Greatwall (Gwl) and Cyclin B (Cyc B) mitotic markers. Endogeneous Gwl-phosphorylation and Cyc B maintenance were monitored for each extract at different times: T0 (immediately after extract preparation), T+1h (corresponding to the 1 h exposure of cells with egg extract) and T+2h to validate the stability of MII-stage extract. A control of mitotic exit corresponding to MII-late stage was also included for each extract, consisting in activation with Ca^2+^ (0.8mM) up to 2 h. Doted rectangle on the right of the figure: 4 pictures of the original blots cropped for figure construction. Dotted rectangle below the figure: pictures at other exposure time and other contrast choices from the original blots.

In the figure, note the existence of a strong phosphorylation differential of the Gwl marker between MII and MII-late stage extracts. MII-stage extracts were characterized by a stable phosphorylation of Gwl (Gwl-P) up to 2 h which was not observed in MII-late stage extracts. On the other hand, Cyc B was stable in the MII stage up to 2 h and degraded in the MII-late stage after 1 h incubation.

The profile of MII and MII-late stage egg extracts were representative of the extracts used to treat cells (T1 - T3 samples and T4 – T7, respectively).

**Supplementary Table S2:** list of the differentially expressed genes between egg extract-treated and control cells. Gene files correspond to cluster-I and cluster-II.

| **CUST number** | **Ensembl ID** | **NCBI ID** | **Gene Symbol** |  | **Sequence (5'- 3')** |
| --- | --- | --- | --- | --- | --- |
| CUST_31048_PI444006311 | ENSCART00000029858 | XM_026276990 | ***bambia*** | Forward | CTT-TGT-GCG-ATG-GCT-GTT-C |
|  |  |  |  | Reverse | GTA-AGA-GGC-GAG-TTT-GTG-TTA |
| CUST_37040_PI444006311 | ENSCART00000091838 | XM_026235311 | ***smad7-1*** | Forward | TGG-AGG-AAC-GGC-GTA-TTC |
|  |  |  |  | Reverse | GGA-GTA-GAG-GCG-CCC-AAC-A |
| CUST_18945_PI444006311 | ENSCART00000021038 | XM_026196925 | ***smad7-2*** | Forward | TGG-AGG-AAC-GGC-GTA-TTC |
|  |  |  |  | Reverse | GGA-GTA-TAG-GCG-CCC-AAC-G |
| CUST_38619_PI444006311 | ENSCART00000152039 | XM_026238759 | ***dusp6-1*** | Forward | GAT-TCC-CAT-CTC-CGA-TCA-CTG |
|  |  |  |  | Reverse | GTT-ACT-GTG-ACG-GAA-CGG-CTA |
| CUST_21745_PI444006311 | ENSCART00000102640 | XM_026202466 | ***dusp6-2*** | Forward | GAT-TCC-CAT-CTC-CGA-TCA-CTG |
|  |  |  |  | Reverse | GTA-ACT-GTG-ACC-GAA-CGG-CTG |
| CUST_10710_PI444006311 | ENSCART00000039124 | _ | ***zeb1b*** | Forward | AAC-GAC-GCT-CCA-CAG-AAG-AAC-A |
|  |  |  |  | Reverse | GCC-CTG-AGT-CTG-AGT-CTG-TC |
| CUST_31093_PI444006311 | ENSCART00000119459 | XM_026259485 | ***mmp9*** | Forward | AAG-ACT-ATG-ACA-AGG-ACA-AGA-T |
|  |  |  |  | Reverse | CTG-TAG-TGC-ATT-TGT-CAT-ATT-TCA-C |
| CUST_629_PI444006311 | ENSCART00000125717 | XM_026261073 | ***fn1b*** | Forward | CAG-TCA-GCG-GAG-GAG-TGG-AGA-G |
|  |  |  |  | Reverse | GAG-ACG-ATA-ACC-TGT-GAC-CTG |
| CUST_3142_PI444006311 | ENSCART00000010729 | XM_026217414 | ***notum1a*** | Forward | GAA-GGA-CAG-TGG-CGT-TAT-AT |
|  |  |  |  | Reverse | GAC-GTG-CCT-TTG-ACT-TGA-AC |
| CUST_28668_PI444006311 | ENSCART00000080640 | XM_026217319 | ***frzb*** | Forward | TAT-AAT-TCT-GGG-TGT-CTG-TGT-CCA |
|  |  |  |  | Reverse | CCG-TGC-CAA-TTT-ATC-TTT-CCA-CTT-CT |

**Supplementary Table S3:** **Real time quantitative PCR primer list**

Extension -1 or -2 in gene symbols corresponds to duplicated genes in goldfish

**
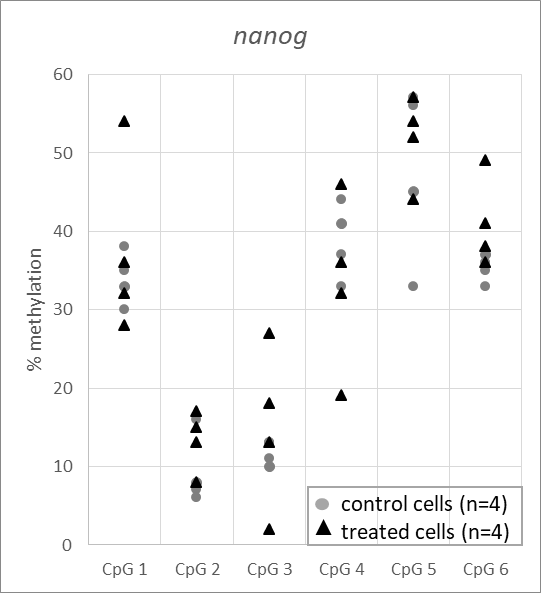
**

**
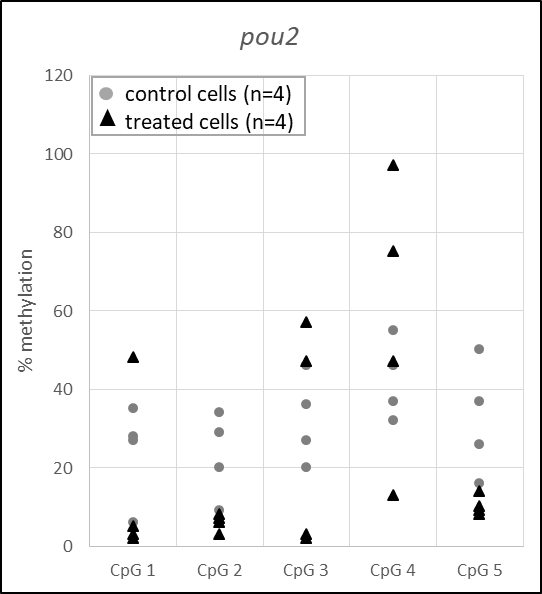
**

**Supplementary Fig. S3:** DNA methylation status of several CpG sites in the promoter regions of *nanog* and *pou2* genes in relation to cell treatment. For *nanog*, CpG1, 2, 3, 4, 5, 6 are located respectively at the sites -324, -270, -217, -164, -134, -122 upstream of the putative transcription start site. Their average methylation status is 27.4 ± 3.3 % (control) and 32.0 ± 2.6 % (treated). For pou2, CpG1, 2, 3, 4, 5 are located respectively at the sites 96, 113, 178, 181, 212 downstream of the putative transcription start site (See Depince et al, 2021 for details). Their average methylation status is 30.8 ± 8.8 % (control) and 22.8 ± 3.0 % (treated). No significant effect of the treatment was observed on these CpG sites.

**A. B.**


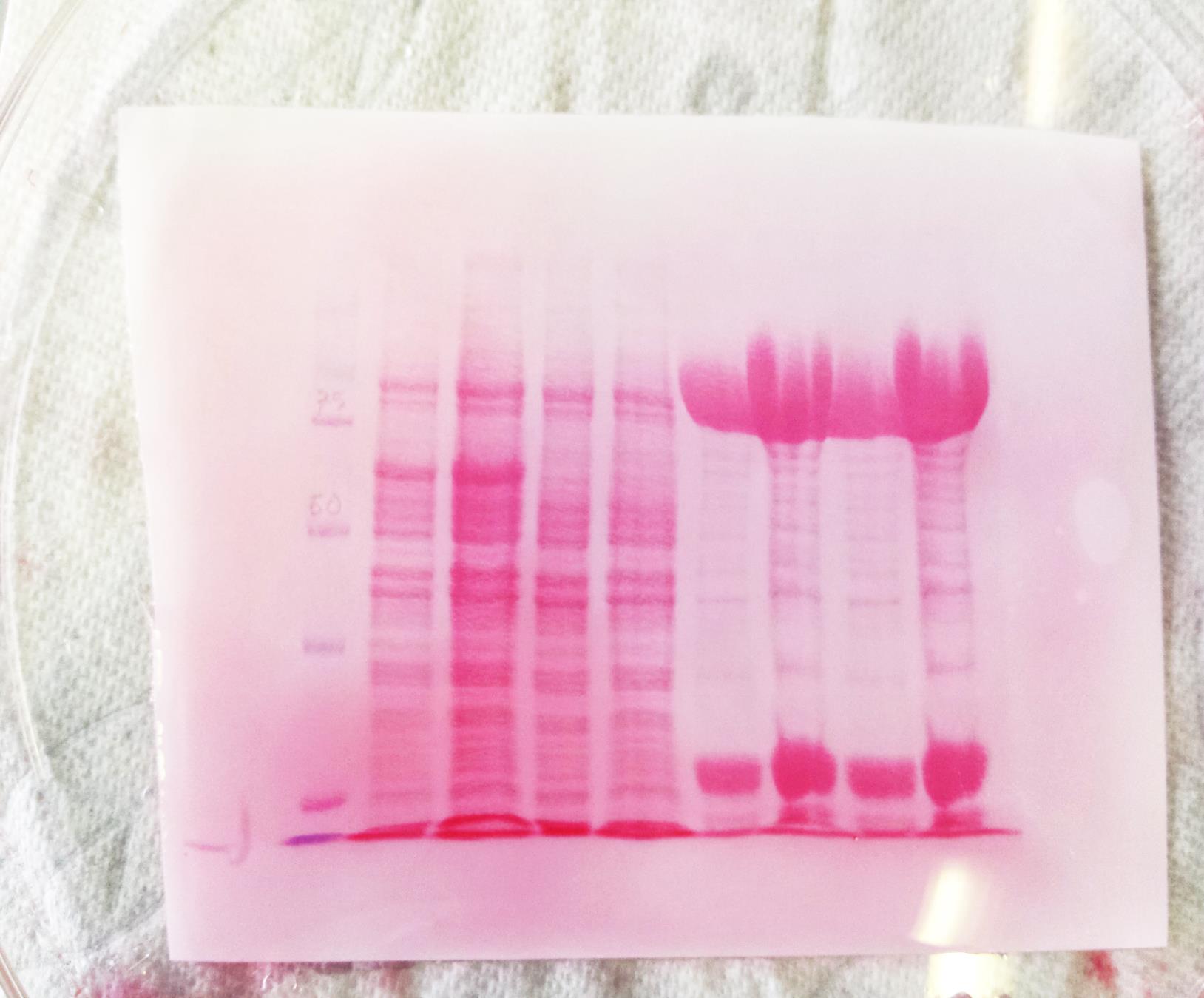


50 _

37 _

25 _

75 _

100 _

***Xenopus egg extract*** ***Goldfish egg extract***

Size

kDa

20µg 40µg

20µg 40µg

20µg 40µg

20µg 40µg


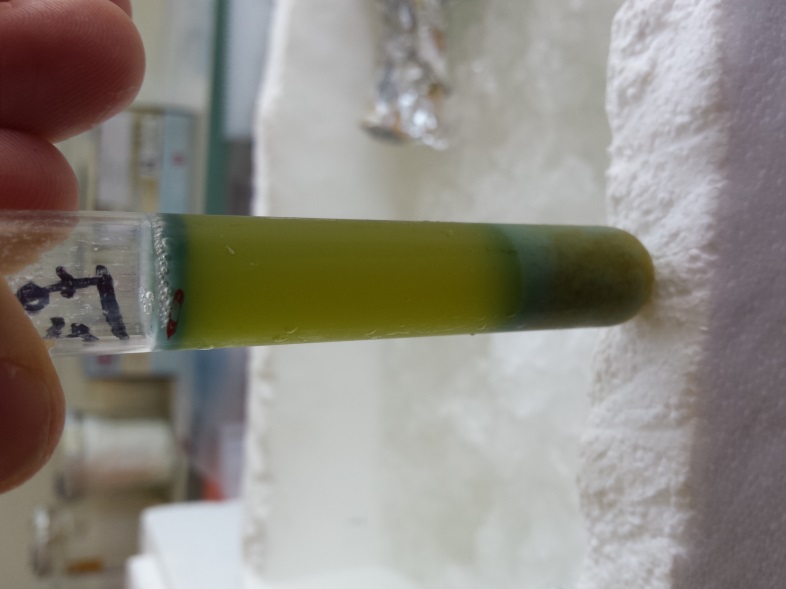

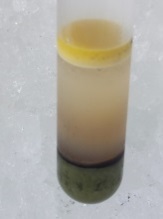


*Golfish egg extract*

*Xenopus*

**Supplementary Fig. S4:** Comparison of *Xenopus* and goldfish egg extracts. A. Egg extracts were prepared according to Chenais et al, 2019 (ref [30] in the main article); B. SDS-PAGE 10 % electrophoresis allowed protein separation (coloration red ponceau). Black arrows: vitellogenins over-represented in the goldfish egg extract.

**Supplementary Methods:**

*Cell culture*

All cell culture procedure has been described in detail in Chenais 2019. The whole fin was minced and digested for 30 min with 2 mg/mL collagenase (Sigma C2674) in Leibovitz’s L15 culture medium (Sigma L5520) supplemented with 5 mM Hepes, 2 mM NaHCO3 2 mM, 100 µg/mL gentamycin, 2.5 µg/mL amphotericin B (osmolality 290 mOsm/kg, pH 7.3) and 10% fetal calf serum, 2mM L-glutamine, 1% non-essential amino acids and 1% sodium pyruvate. Cells were plated in 6-well plates in growth L15 culture medium. After 24 hours, the supernatant enriched with mesenchymal cells, adhering more slowly than epithelial cells, was collected. These cells have previously been shown to be the most suitable for nuclear transfer (Chenais 2014, 2015). After filtration and washing, the cells derived from supernatant were seeded at 0.2.10^6^ cells in 24 well plates on 1.3 cm^2^ glass coverslips and cultured in L15 medium for 2 days (about 80% confluence) until treatment experiments.

*RTqPCR analysis*

All steps of RT and qPCR were described in Chenais 2015. Briefly, 500 ng of total RNA was used to reverse transcription (RT) with the GoScript®™ Reverse Transcriptase System (A5001, Promega). Control reactions (RT- controls) were performed without the GoScript reverse transcriptase. All samples and controls were diluted 1/15 prior to qPCR. The primer sequences, concentrations and annealing temperatures used for *col1a1a, nanog, pou2, sox2, c-myca1* and *c-myca2*, cDNA detection are described in Chenais 2015. The qPCR reactions were carried out in duplicates from 5 µL of cDNA samples or negative controls, 6 µL of the SYBR® Green Master Mix (Applied Biosystems) and 1 µL of reverse and forward primers mix. PCR were run on a StepOne real-Time PCR System (Applied Biosystems). Specificity of the PCR product was checked for each primer set and samples from the melting curve analysis.

*Gene candidate DNA methylation analysis*

Total extracted DNA was purified using the Genomic DNA Purification and Concentration Kit (Zymo Research, D4010) and quantified using the QubitTM dsDNA HS Assay Kit (Q32851, Invitrogen) on the QubitTM 4 Fluorometer. DNA (10ng) was treated with bisulphite using the EZ DNA Methylation-Gold kit (Zymo Research, D5006). The bisulphited DNA was stored in a final volume of 12 µL at -20°C and analysed within 3 months of conversion. PCR reactions were performed to amplify the bisulphite sequences of regions of interest (promoter regions previously selected by the team), containing marker CpG sites, in each sample. Each reaction was performed in a final volume of 25 µL, using 2 µL of bisulphited DNA, the Advantage® 2 Polymerase enzyme (100X, Takara, 639202), the Advantage® 2 PCR buffer (10X, Takara, 639137), 0.2 µL of dNTPs (25 mM each, PROMEGA, U1330), 2 µL of primers (5 µM each, forward and reverse) and sterile water free of nucleases (PROMEGA, P1193). The reaction was carried out after denaturation of the components for 2 min at 94°C. The number of cycles as well as the temperature of hybridization of the primers is referenced in Depince et al., 2021.

A second PCR serie (PCR nests) was carried out, using second pairs of primers selected from the ends of the amplicons generated by the first pairs of primers. Reverse primers of this nested PCR were coupled with biotin at their 5'end, in order to biotinylate the strands of amplified DNA, which is necessary for the subsequent sequencing steps. Each nested PCR reaction was performed in a final 50 µL volume, in duplicate, with 2 µL of products from the first PCR diluted 1/20th and following the same protocol as for the first reactions. Information about cycle number, hybridization temperature of the nested PCR primers is also given Depince et al., 2021. The nested PCR duplicates were then pooled and stored at -20°C. The quality of the nested PCR products (size and presence of a single amplicon) was tested by electrophoresis on a 2% agarose gel (Eurogenetec, EP-0010-05) including fluorescent nucleic acid intercalant (10,000X Red Nucleic Acid Gel Stain, Biotum).
